# Efficacy and safety of Chinese herbal medicine in the treatment of chronic pruritus: A systematic review and meta-analysis of randomized controlled trials

**DOI:** 10.3389/fphar.2022.1029949

**Published:** 2023-01-12

**Authors:** Jie Wang, Yuhang Chen, Xinwei Yang, Jianli Huang, Yihua Xu, Wei Wei, Xianbo Wu

**Affiliations:** ^1^ The First Affiliated Hospital of Guizhou University of Traditional Chinese Medicine, Guiyang, Guizhou, China; ^2^ The Second Clinical Medical College of Beijing University of Chinese Medicine, Beijing, China; ^3^ School of Sports Medicine and Health, Chengdu Sport University, Chengdu, Sichuan, China; ^4^ School of Basic Medical Sciences, Chengdu University of Traditional Chinese Medicine, Chengdu, Sichuan, China

**Keywords:** Chinese herbal medicine (CHM), chronic pruritus (CP), pruritus degree, systematic review, meta-analysis

## Abstract

**Background:** Chronic pruritus (CP) is a common and aggravating symptom associated with skin and systemic diseases. Although clinical reports suggest that Chinese herbal medicine (CHM) is safe and effective in Chronic pruritus treatment, evidence to prove it is lacking. Therefore, in this review, we evaluated the therapeutic effects and safety of Chinese herbal medicine for the treatment of Chronic pruritus.

**Methods:** Nine databases were searched for relevant randomized controlled trials (RCTs) from the inception of the database to 20 April 2022. The randomized controlled trials that compared the treatment of Chinese herbal medicine or a combination of Chinese herbal medicine and conventional western medicine treatment (WM) with western medicine treatment intervention for patients with Chronic pruritus were selected. We evaluated the effects of treatment with Chinese herbal medicine on the degree of pruritus, the Dermatology Life Quality Index (DLQI) score, response rate, recurrence rate, and incidence of adverse events in patients with Chronic pruritus. The risk of bias in each trial was evaluated using the Cochrane Collaboration tool. The RevMan software (version 5.3) was used for performing meta-analyses to determine the comparative effects.

**Results:** Twenty-four randomized controlled trials were included, compared with placebo, moderate-quality evidence from one study showed that Chinese herbal medicine was associated with reduced visual analogue scale (VAS) (MD: −2.08; 95% CI = −2.34 to −1.82). Compared with western medicine treatment, low-to moderate-quality evidence from 8 studies indicated that Chinese herbal medicine was associated with reduced visual analogue scale, 4 studies indicated that Chinese herbal medicine was associated with reduced Dermatology Life Quality Index (MD = −1.80, 95% CI = −2.98 to −.62), and 7 studies indicated that Chinese herbal medicine was associated with improved Effective rate (RR: 1.26; 95% CI = 1.19–1.34). Compared with combination of Chinese herbal medicine and western medicine treatment, 16 studies indicated that Chinese herbal medicine was associated with reduced visual analogue scale, 4 studies indicated that Chinese herbal medicine was associated with reduced Dermatology Life Quality Index (MD = −2.37, 95% CI = −2.61 to −2.13), and 13 studies indicated that Chinese herbal medicine was associated with improved Effective rate (RR: 1.28; 95% CI = 1.21–1.36). No significant difference in the occurrence of adverse events in using Chinese herbal medicine or western medicine treatment was reported.

**Conclusion:** The efficacy of Chinese herbal medicine used with or without western medicine treatment was better than western medicine treatment in treating chronic pruritus. However, only a few good studies are available regarding Chronic pruritus, and thus, high-quality studies are necessary to validate the conclusions of this study.

## 1 Introduction

Chronic pruritus (CP) is an unpleasant sensation that induces an urge to scratch and lasts for at least 6 weeks (Rajagopalan et al., 2017; Villa-Arango et al., 2019). It is often accompanied by skin diseases [e.g., psoriasis, atopic dermatitis (AD), and lichen planus] and systemic diseases (e.g., end-stage renal disease, diabetes, hypothyroidism, chronic hepatobiliary disease, and malignancy) (Twycross et al., 2003; Ständer et al., 2007; Olek-Hrab et al., 2016; Weisshaar et al., 2019). Several population-based studies have suggested that one in five individuals in the general population experience CP at least once in their lifetime, with a 12-month incidence of 7% (Matterne et al., 2011). The prevalence of CP in the general adult population is approximately 13.5% (Rajagopalan et al., 2017). In patient populations, the incidence of CP varies depending on its underlying etiology, ranging from 25% in hemodialysis patients (Weiss et al., 2016) to 100% in patients with skin conditions, such as urticaria and AD (Szepietowski et al., 2002; Szepietowski et al., 2004). CP can lead to sleep disturbance, fatigue, inability to work, anxiety, and depression, resulting in a considerable decline in health-related quality of life (Schneider et al., 2006; Steinke et al., 2018; Stumpf et al., 2018; Schneider et al., 2022). Additionally, CP imposes a significant burden on society by increasing healthcare costs and posing treatment challenges.

The cause of CP is extremely complicated and includes dermatological, systemic, neurological, psychiatric, mixed, or unknown factors (Kretzmer et al., 2008; Matterne et al., 2013; Shive et al., 2013; Hay et al., 2014; Pereira et al., 2016). CP is a challenging condition to manage due to its extremely complicated aetiology. The treatment of CP mainly includes the treatment of the underlying disease and topical treatment. Conventional western medicine treatment (WM) (Andrade et al., 2020) includes emollient creams, cooling lotions, topical corticosteroids, topical antidepressants, systemic antihistamines, systemic antidepressants, systemic anticonvulsants, and phototherapy, as well as, symptomatic and supportive care. However, the commonly (Hay et al., 2014) used treatment methods have limited efficacy and might be associated with significant side effects. Therefore, patients often experience severe, long-term itching without improvement, which exacerbates the negative effects on the quality of life and psychosomatic responses (Dalgard et al., 2020). Therefore, alternative strategies for treating CP need to be investigated.

Chinese herbal medicine (CHM) is an essential part of monotherapy or substitute supplementary treatment of CP and has been used in China for many years. The treatment of itching by administering CHM (mainly orally), CHM fumigation, external washing, acupoint therapy, etc., can effectively relieve itching. Many clinical and experimental studies have confirmed the effectiveness of CHM in the treatment of CP (Bedi and Shenefelt, 2002; Xue et al., 2019; Lu et al., 2021). For example, Qinzhuliangxue decoction has anti-inflammatory effects, and it increases the threshold of pruritus caused by histamine phosphate, which is effective in treating CP caused by specific eczema (Ma et al., 2020). Turmeric has anti-inflammatory and high-sensitivity C-reactive protein–lowering effects and can be used for treating uremic pruritus (Pakfetrat et al., 2014). Animal experiments have shown that administering Huanglian Jiedu decoction can treat AD by regulating the antigen presentation function of dendritic cells, weakening T-lymphocyte activation, and subsequently exerting anti-inflammatory and anti-pruritus effects (Xu et al., 2021). Although several studies have treated CP with CHM or a combination of CHM and WM, systematic analyses and evidence synthesis of CHM treatment on CP are limited. Therefore, new evidence-synthesis methods need to be developed in this field. In this systematic review, we summarized and evaluated studies on the efficacy and safety of using CHM for monotherapy or adjuvant therapy in the treatment of CP to promote its clinical application.

## 2 Materials and methods

The review protocol was registered on the International Platform for the Registration of Systematic Review and Meta-Analysis Schemes (INPLASY202260103), and it is presented according to the Preferred Reporting Items for Systematic Review and Meta-Analysis (PRISMA) Statement (Liberati et al., 2009).

### 2.1 Search strategies

Nine electronic databases were searched for relevant studies from the date of the inception of the database to 20 April 2022. The databases searched were PubMed, Embase, Web of Science, Cochrane, Chinese Biological Medicine, China National Knowledge Infrastructure, the Chinese Scientific Journal Database, the Wanfang database, and Clinical Trials.gov. Publications of all languages were accepted. Relevant studies were retrieved using Medical Subject Heading terms or keywords combined with free text words, such as chronic pruritus, pruritus, traditional Chinese medicine (TCM), Chinese herbal medicine, randomized control, and random. These keywords were modified according to the needs of different databases. The PubMed search strategies are shown in Table 1.

**TABLE 1:** PubMed search strategy.


#1: “Drugs, Chinese Herbal” [Mesh]
#2: (((((((Chinese Drugs [Title/Abstract]) OR (Chinese Plant [Title/Abstract])) OR (Chinese Herbal Drugs [Title/Abstract])) OR (herbal drugs [Title/Abstract])) OR (Chinese [Title/Abstract])) OR (plant extracts Chinese [Title/Abstract])) OR (Chinese plant extracts [Title/Abstract])) OR (extracts, Chinese plant [Title/Abstract])
#3: 1# OR 2#
#4: “Pruritus” [Mesh]
#5: (((Itching [Title/Abstract]) OR (Chronic pruritus [Title/Abstract])) OR (itch [Title/Abstract])) OR (Chronic itch [Title/Abstract])
#6: 4# OR 5#
#7: “Randomized Controlled Trials as Topic” [Mesh]
#8: (“Randomized Controlled Trials as Topic” [Mesh]) OR (((Clinical Trials, randomized [Title/Abstract]) OR (Trials, Randomized Clinical [Title/Abstract])) OR (Controlled Clinical Trials, Randomized [Title/Abstract]))
#9: 7# OR 8#
#10: 3# OR 6# OR 9#

### 2.2 Inclusion and exclusion criteria

We evaluated the efficacy and safety of CHM for treating CP. The inclusion criteria for the studies were as follows: (Rajagopalan et al., 2017) a randomized controlled trial (RCT) was performed with or without blinding; (Villa-Arango et al., 2019) the participants were diagnosed with chronic systemic pruritus (pruritus duration >6 weeks) (Ständer et al., 2007); (Ständer et al., 2007) the participants were not restricted to a specific age group, gender, race, disease duration, or concomitant disease; (Weisshaar et al., 2019) the experimental group was treated with oral, external, or a combination of oral and external CHM, irrespective of the medicinal form used (e.g., proprietary Chinese medicine, Chinese herbal decoction, granules, capsules, tablets, pills, or injections), whereas, the control group was treated with a placebo or conventional Western medicine (WM); (Olek-Hrab et al., 2016) the pruritus index (using the visual analogue scale [VAS] (Furue et al., 2013)) was reported in the study.

The exclusion criteria for the studies were as follows: (Rajagopalan et al., 2017) the participants were not diagnosed with CP, or the study did not mention that the duration of pruritus was >6 weeks; (Villa-Arango et al., 2019) the RCT did not use the VAS to assess pruritus; (Ständer et al., 2007) the control group was administered other TCM interventions (such as CHM, acupuncture, or massage) other than WM. The studies were screened based on the selection criteria by two independent reviewers (WJ and CYH), and a third reviewer (WXB) resolved any discrepancy that might have occurred between the assessments of the two reviewers.

### 2.3 Types of outcome measures

The main outcome measure was the pruritus index evaluated using VAS. VAS consists of a 10-cm line indicated with points 0 to 10 (0 = no itching, 10 = worst imaginable itching) on which patients indicate pruritus intensity by marking the point that corresponds to the severity of their pruritus (Furue et al., 2013). Secondary outcomes included the Dermatology Life Quality Index (DLQI) score (Finlay and Khan, 1994), effective rate, recurrence rate, and adverse effect rate.

### 2.4 Data extraction and quality assessment

After retrieving the articles, the documents were managed, and duplicates were removed using the document management software Endnote X9. Based on the inclusion and exclusion criteria, two reviewers (WJ and CYH) independently screened the titles and abstracts to select relevant studies, and then, they screened the full texts for the final selection. Additionally, the name of the authors, the year of publication, sample size, the sex and age of the participants, details of the intervention, outcome measures, and adverse reactions were independently extracted by two reviewers (WJ and CYH) using a pre-defined data collection form. Then, the extracted data were cross-checked for accuracy by two other reviewers (HJL and YXW). In case information in the included articles was not clear, one of the reviewers (HJL) contacted the authors of the specific study through telephone or email for clarification. The included articles were evaluated by two reviewers (WJ and CYH) using ROB2.0. The six dimensions included random isolation process, deviations from intended interventions, missing outcome data, measurement of the outcome, selection of the reported result, and overall bias. If disagreement occurred between the reviewers, discussions were held with the two other reviewers (XYH and WW) to arrive at a consensus.

### 2.5 Evidence synthesis and statistical analysis

Cochrane systematic review software Review Manager 5.3 and Stata 14.0 were used to analyze the data statistically. The quality of evidence was summarized and graded using the Grading of Recommendations Assessment, Development, and Evaluation (GRADE) methodology. A table was created to summarize the results using the GRADE profiler 3.6.1 software. Risk ratios (RRs) with 95% confidence intervals (CIs) were used to evaluate dichotomous data, continuous data, mean difference (MD), and standard mean difference. A fixed-effects model was used if the data were homogeneous (Cochrane Q, *p* > .1, I^2^ < 50%), and a random-effects model was used if the data were heterogeneous. *p*-values <.05 were considered to be statistically significant. When a certain level of heterogeneity was observed, and we had sufficient studies, subgroup analyses were performed to account for the heterogeneity. Funnel plots were used to assess publication bias when at least 10 trials were available.

## 3 Results

### 3.1 Description of the included studies

**FIGURE 1:**
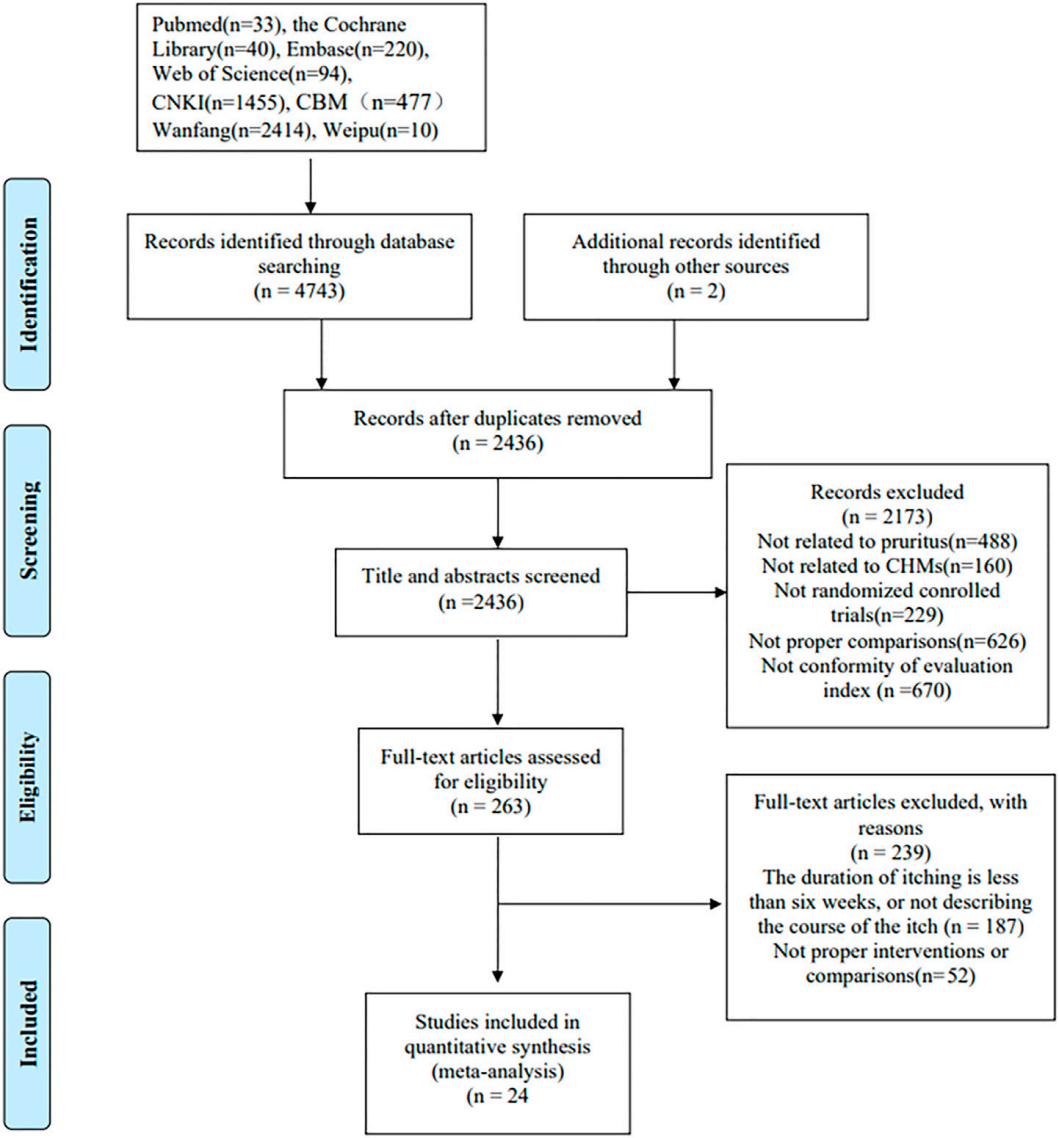
A flow diagram of the study selection process. CNKI, China National Knowledge Infrastructure; CBM, Chinese Biological Medicine; CHM, Chinese herbal medicine.

Following our search strategy, 4,745 articles were initially identified, and 2,436 articles were selected after removing duplicates. After initially screening the titles and abstracts for inclusion and exclusion criteria, 2,173 articles were excluded. The remaining 263 studies were thoroughly reviewed, and 239 articles were removed for various reasons. Finally, 24 eligible RCTs (Ying Yang et al., 2006; Mehrbani et al., 2015; Wei and Li, 2017; Yine and Li, 2017; Yu Zhou et al., 2017; Hu and Feng, 2018; Liu, 2018; Ma, 2018; Qi, 2018; Tianming Ma and Liu, 2018; Yang et al., 2018; Zhang, 2018; Hanhua Cao et al., 2019; Li, 2019; Liu, 2019; QIi, 2019; Qing Wu et al., 2020; Rui Tao et al., 2020; Xinwei Guo et al., 2020; Yu, 2020; Bin Zhao, 2021; Lan, 2021; Liu, 2021; Tianhua Quan et al., 2021) consisting of 2,313 participants were included in the meta-analysis. The study selection process is presented in Figure 1, the characteristics of the included trials are presented in Table 2, and the characteristics of the included TCM are presented in Table 3; Supplementary Table S1.

**TABLE 2:** Characteristics of the included studies.

Study ID	Conditions	Sample size		Course of disease		Mean age (year)		Male/Female		Intervention		The course of treatment (m = month)	Outcomes
		Experimental	Control	Experimental	Control	Experimental	Control	Experimental	Control	Experimental	Control		
Li (2019)	chronic urticaria	38	38	39.52 ± 4.76w	39.55 ± 4.81w	45.08 ± 3.54	45.09 ± 3.52	22/16	21/17	Loratadine dispersive tablet + Xiaoyang Decoction	Loratadine dispersive tablet	2 weeks	①
Zhang (2018)	psoriasis	28	28	12.1 ± 10.4 m	12.4 ± 11.4 m	41.3 ± 15.2	45.5 ± 11.6	18/12	16/14	Vaseline + traditional Chinese medicine bath	Vaseline	3 weeks	①③
Qi (2018)	eczema	42	38	5.72 ± 2.46 m	5.36 ± 2.61 m	38.19 ± 8.74	37.45 ± 8.52	19/23	17/21	Jianpi Qushi Decoction + Mometasone Furoate Cream	Xitirizine Hydrochloride Tablets + Mometasone Furoate Cream	1 month	①③⑤
Liu (2018)	neurodermatitis	32	31	27.7 ± 15.4 m	29.1 ± 16.3 m	46.8 ± 15.6	45.3 ± 14.5	19/16	17/18	Chinese herbal washing externally and drinking internally	compound dexamethasone cream + zinc oxide-sulfur ointment + loratadine tablets	28 days	①②③
Wei and Li, 2017	neurodermatitis	74	73	29.5 ± 16. 3 m	31.6 ± 19. 2 m	46.2 ± 15.3	45.8 ± 14.7	38/42	43/37	Qingshi Zhiyang ointment + Modified Xiaofeng	Mometasic Fumarate cream + loratadine tablets	21 days	①②③
										Zhiyang Decoction			
Tianhua Quan (2021)	chronic urticaria	30	30	3.31 ± 2.16y	3.08 ± 2.20y	36.23 ± 13.25	36.23 ± 13.25	13/17	12/18	Pingwei Xiaozhen Decoction + olopatadine hydrochloride tablets	olopatadine hydrochloride tablets	4 weeks	①③④⑤
Bin Zhao (2021)	chronic urticaria	58	58	3.87 ± .78y	3.66 ± .71y	41.25 ± 8.27	41.37 ± 8.24	28/30	27/31	Xiaoxun decoction + desloratadine citrate disodium tablets	desloratadine citrate disodium tablets	3 weeks	①②③④
Yu (2020)	chronic eczema	160	160	11.36 ± 2.47 m	11.58 ± 2.64 m	39.25 ± 6.87	39.79 ± 7.02	84/76	86/74	Traditional Chinese Medicine fumigation	Mizolastine Sustained Release Tablets + Mometasone Furoate Cream	20 days	①③
Hanhua Cao (2019)	pruritus in Hemodialysis Patients	30	30	36.45 ± 10.72 m	35.57 ± 10.28 m	51.83 ± 9.22	52.76 ± 10.24	16/14	15/15	hemoperfusion + gabapentin + Chinese herbal fumigation	hemoperfusion + gabapentin	60 days	①③
Liu (2019)	psoriasis	55	55	3.9 ± 2.6y	4.3 ± 2.5y	39.19 ± 11.76	37.2 ± 9.64	31/24	28/27	Traditional Chinese Medicine No.2 Prescription + Jianpi Jiedu Decoction	Awei A Capsule + calcipotrio ointment	12 weeks	①②③⑤
Qii (2019)	eczema	75	75	3.26 ± 1.47y	3.27 ± 1.49y	44.16 ± 9.16	44.23 ± 9.21	38/37	39/36	Jianpi Huashi decoction + routine treatment + hydrocortisone	routine treatment + hydrocortisone	8 weeks	①②
Yu Zhou (2017)	psoriasis	53	53	3.5 ± 0.5y	3.7 ± 0.6y	36.5 ± 8.4	35.5 ± 8.0	31/22	33/20	Vl000L phototherapy machine treatment + herbal bath of Liangxue Zhiyang decoction	Vl000L phototherapy machine treatment	8 weeks	①②③⑤
Xiaojing Yang (2018)	allergic dermatitis	40	40	1.03 ± .38y	1.10 ± .47y	34.33 ± 6.97	33.94 ± 6.28	16/24	15/25	White Tiger Decoction + Dexamethasone Acetate Ointment + Desloratadine Tablets	Dexamethasone Acetate Ointment + Desloratadine Tablets	4 weeks	①③
Liu (2021)	psoriasis	32	32	7.68 ± 5.02y	8.12 ± 4.11y	36.89 ± 7.56	37.55 ± 8.03	18/14	16/16	Calcipotriol ointment + Danggui Sini decoction	Calcipotriol ointment	8 weeks	①③⑤
Tianming Ma (2018)	atopic dermatitis	43	36	15.7 ± 8.2y	14.9 ± 7.9y	27.5 ± 6.3	26.3 ± 7.1	28/15	24/12	Compound fish liver oil oxidation ointment + tacrolimus ointment + Kushen Qufeng pills	Compound fish liver oil oxidation ointment + tacrolimus ointment	4 weeks	①②③⑤
Yine and Feng (2017)	plaque psoriasis	70	70	6.41 ± 1.00y	6.49 ± 1.03y	41.02 ± 5.39	40.76 ± 5.32	32/28	39/31	Acitretin + Yangxue Tongluo decoction	Acitretin	8 weeks	①②③⑤
Ying Yang (2007)	atopic dermatitis	32	32	NA	NA	NA	NA	NA	NA	Jianpi Zhiyang Granules	loratadine	4 weeks	①⑤
Saishen Hu (2018)	Senile skin pruritus	44	43	2.01 ± .29y	1.95 ± .36y	7.90 ± 6.07	68.11 ± 5.87	27/17	28/15	Cetirizine + Calamine Liniment + Modified Siwu Decoction Combined with Yangxue Runzao Zhiyang Prescription	Cetirizine + Calamine Liniment	2 weeks	①③⑤
Lan Ding (2021)	Pruritus in Hemodialysis Patients	20	20	NA	NA	NA	NA	NA	NA	Hemodialysis + Ebastine + Yangxue Qufeng External Washing Prescription	Hemodialysis + Ebastine	4 weeks	①③
Ma (2018)	chronic eczema	42	43	2.04 ± .34 m	2.13 ± .49 m	37.21 ± 1.33	36.84 ± 1.48	25/17	28/15	antiallergic treatment + Compound Flumethasone Ointment + Yangxue Zhiyang Decoction	antiallergic treatment + Compound Flumethasone Ointment	14 days	①③
Mehrbani (2015)	atopic dermatitis	24	18	13.45 ± 1.82y	12.77 ± 2.09y	28.62 ± 2.30	24.33 ± 1.50	20/4	16/18	extract of field dodder	placebo	15 days	①⑤
Qing Wu (2020)	psoriasis	39	39	4.82 ± .92y	4.76 ± .88y	42.95 ± 8.06	43.06 ± 8.18	21/18	20/19	compound glycyrrhizin tablets + desonide cream + Qingre Liangxue decoction	compound glycyrrhizin tablets + desonide cream	8 weeks	①③④⑤
Rui Tao (2020)	chronic eczema	36	35	8.16 ± 2.56 m	8.20 ± 3.02 m	56.23 ± 12.13	55.34 ± 11.28	24/12	22/13	conventional hypoglycemic treatment + triamcinolone econazole cream + Jiangtang Huoxue Prescription combined with Xiaofeng Powder	conventional hypoglycemic treatment + triamcinolone econazole cream + loratadine dispersible tablets	4 weeks	①②④⑤
Xinwei Guo (2020)	atopic dermatitis	57	57	15.07 ± 5.52y	15.86 ± 6.86y	24.42 ± 6.01	26.86 ± 6.93	30/27	29/28	Modified Chushi Weiling Decoction + vitamin E cream	loratadine tablets + vitamin E cream	8 weeks	①②⑤

①visual analogue scale (VAS); ②dermatology life-quality index (DLQI); ③Effective rate; ④Recurrence rate; ⑤Adverse effect rate.

**TABLE 3:** Ingredients of CHM in the included studies.

Study	Prescription name	Ingredients of herb prescription	Preparations
Chan Li (2019)	Xiaoyang Decoction	huangqi 20 g, shenyao 20 g, baixianpi 15 g, baishao15 g, niubangzi15 g, danggui 10 g, fangfeng 10 g, muzui 10 g, xuchangqing 10 g, gancao 5 g	Decoction
Jing Zhang (2018)	traditional Chinese medicine bath	danshen 50 g, honghua 20 g, shengdi 50 g, digupi 50 g, kushen 40 g, baixianpi 40 g, difuzin 40 g, shechuangzi 30 g, baihuasheshecao 30 g	—
Fengjuan Wang (2018)	Jianpi Qushi Decoction	dansheng 20 g, caobaizhu 15 g, fuling 10 g, mudanpi 10 g, fangfeng10 g, zexie 10 g, chenpi 10 g, chishao10 g, difuzi 15 g, shanyao 10 g, huashi 10 g, baixianpi 10 g, yiyiren 10 g, zhiganzao 6 g	Decoction
Tianming Ma (1) (2018)	Chinese herbal washing externally and drinking internally	shengdihuang 20 g, danggui 15 g, maidong 20 g, baishao 30 g, mudanpi 20 g, kushen 20 g, jili 20 g, fangfeng 15 g, baiixanpi 20 g	Decoction
Wei Li (2017)	Qingshi Zhiyang ointment + Modified Xiaofeng Zhiyang Decoction	qingdai, luganshi, danshigao, huashi, kushen, huangbai, bingpian, Olive oil, vaseline + quanxie 3 g, chanyi 10 g, wushaoshe 10 g, lufengfang 10 g, baixianpi 15 g, xuzhangqing 15 g, baishao 20 g, chuanxiong 10 g, danshen20 g	ointment, Decoction
Tianhua Quan (2021)	Pingwei Xiaozhen Decoction	jingjie 15 g, fangfeng 15 g, dahuang 6 g, mangxiao 9 g, chuanxiong 10 g, danggui 20 g, baishao 15 g, huangqin 15 g, jiegeng 12 g, gancao 10 g, baizhu 1 5g, shigao 20 g, yinchaihu 12 g, wuweizi 9 g, wumei 9 g, baixianpi 15 g, cijili 15 g	Decoction
Bin Zhao (2021)	Xiaoxun decoction	jingjie 15 g, fangfeng 15 g, chantui 10 g, baijili 15 g, danggui 6 g, chuanxiong 10 g, chenpi 10 g, fuling10 g, baizhu 10 g, shenggancao 10 g	Decoction
Shengbin Yu (2020)	Traditional Chinese Medicine fumigation	hongzicao 30 g, fangfeng 15 g, difuzi 30 g, shengdi 30 g, huajiao10 g, baixianpi 30 g, shechuangzi 20 g, taoren 30 g, gancao 15 g, chishao 30 g, binpian 5 g, dahuang 30 g, kushen 30 g	CHM fumigation
Hanhua Cao (2019)	Chinese herbal fumigation	fangfeng 15 g, chantui 15 g, baixianpi 50 g, dahuang 30 g, tufuling 30 g, difuzi 30 g, shechuangzi 30 g, baishouwu 30 g, jixueteng30 g, danshen 30 g, chuanxiong 30 g, kushen 10 g, bohe 10 g	CHM fumigation
Pengying Li (2019)	Traditional Chinese Medicine No.2 Prescription + Jianpi Jiedu Decoction	tufuling 30 g, bixie 10 g, fuling 12 g, huangbai 20 g, lianqiao 15 g, chaobaizhu 10 g, baihuasheshecao 30 g, danggui 20 g, danshen 10 g, kushen 10 g, yiyiren 20 g, gancao 10 g + tufuling 30 g, huangbai 30 g, machixian 30 g, lianqiao 30 g, baizhu 30 g, fuling 30 g	Decoction
Shaoqun Qi (2019)	Jianpi Huashi decoction	chaochenpi 10 g, baizhu 10 g, fuling 10 g, danzhuye 10 g, fangfeng 10 g, baixianpi 10 g, cangzhu 10 g, baijili 10 g, chaoyiyiren 15 g, shengmuli 20 g, taizishen 20 g, gancao 6 g	Decoction
Yu Zhou (2017)	Liangxue Zhiyang decoction	danshen 20 g, kushen 12 g, jingjie 12 g, zicao 10 g, fangfeng12 g, chantui 8 g, yejuhua 10 g, shechuangzi 10 g, tufuling 10 g, guizhi 10 g, dazao 12 g, gancao 6 g	CHM bath
Xiaojing Yang (2018)	White Tiger Decoction	baishao 20 g, shigao 20 g, jinyinhua 20 g, xuanshen 20 g, pugongying 20 g, baixianpi 20 g, shengdihuang 25 g, zhimu 15 g, danpi 15 g, wushe 15 g, fangfeng 12 g, danggui 12 g, quanxie 9 g, chaihu 9 g, gancao 9 g	Decoction
Xiaohui Liu (2021)	Danggui Sini decoction	danggui 30 g, shanyao 30 g, chuanxiong 15 g, jixueteng 15 g, zhigancao 15 g, chishao 10 g, baishao 10 g, jingjie 10 g, fangfeng 6 g, cangerzi 6 g, chaihu 6 g, guizhi 6 g, xixin 3 g	Decoction
Tianming Ma (2) (2018)	Kushen Qufeng pills	—	Pill
Yine Song (2017)	Yangxue Tongluo decoction	tufuling 30 g, shenghuaihua 20 g, danggui 15 g, jixueteng 15 g, weilingxian 15 g, fangfeng 15 g, dihuang 15 g, maidong 10 g	Decoction
Ying Yang (2006)	Jianpi Zhiyang Granules	huangqi, baizhu, danggui, heshouwu, shengdi, baishao, chuanxiong, jingjie, fangfeng	Capsule
Saiqian Hu (2018)	Modified Siwu Decoction Combined with Yangxue Runzao Zhiyang Prescription	danggui 10 g, chuanxiong 10 g, shaoyao 10 g, shudihuang 15 g, baixianpi 10 g, fangfeng 10 g, jingjie 10 g, shengdihuang 20 g, tufuling 20 g, difuzi 20 g, nvzhenzi 15 g, kushen 15 g, zhimu 15 g, mohanlian 20 g, huangbai12 g, mudanpi 12 g, shuiniujiaofen10 g	Decoction
Lan Ding (2021)	Yangxue Qufeng External Washing Prescription	danggui 15 g, chishao 10 g, chuanxiong 9 g, danpi 15 g, kushen15 g, tufuling 15 g, baijili1 5g, yejiaoteng 30 g, difuzi 15 g, shechuangzi15 g, jingjie 10 g, fangfeng 10 g, shenggancao 30 g	CHM bath
Yinping Ma (2018)	Yangxue Zhiyang Decoction	shengdihuang 25 g, shudihuang 25 g, tiandong 25 g, maidong 25 g, mudanpi 20 g, jingjie 20 g, danggui 10 g, baijili 10 g, chantui10 g, gancao 10 g	Decoction
Mehrzad Mehrbani	extract of field dodder	dodder seed	
Qing Wu (2020)	Qingre Liangxue decoction	lianqiao 30 g, zicao 25 g, shuiniujiao15 g, jinyinhua 15 g, danpi12 g, shengdi 12 g, huangqin 10 g, chishao10 g, lingxiaohua 10 g, gancao 9 g	Decoction
Rui Tao (2020)	Jiangtang Huoxue Prescription combined with Xiaofeng Powder	cangzhu 15 g, xuanshen 20 g, gegen 15 g, danshen 20 g, huangqi 20 g, shengdihuang 20 g, muxiang 10 g, danggui 10 g, yimucao15 g, baishao 15 g, chuanxiong 10g, fangfeng 10 g, chantui 10 g, zhimu 10 g, kushen 6 g, mutong 6 g, jingjie 10 g, niubangzi 10 g, shigao 15 g, gancao 6 g	Decoction
Xinwei Guo (2020)	Modified Chushi Weiling Decoction	cangzhu 6 g, houpu 6 g, chenpi 9 g, huashi 12 g, chaobaizhu 12 g, zhuling 12 g, chaohuangbai 12 g, chaozhike 9 g, zexie 9 g, chiling 12 g, zhigancao 9 g	Decoction

All studies were published between 2007 and 2022. The number of participants in the studies varied from 42 to 300, and the treatment duration varied from 14 to 60 days. The participants had different types of disease along with CP. Among the 24 studies, the experimental groups of eight studies (Ying Yang et al., 2006; Wei and Li, 2017; Liu, 2018; Qi, 2018; Liu, 2019; Rui Tao et al., 2020; Xinwei Guo et al., 2020; Yu, 2020) used CHM treatment, of which 5 (Ying Yang et al., 2006; Wei and Li, 2017; Liu, 2018; Rui Tao et al., 2020; Yu, 2020) had a treatment period of ≤4 weeks and three studies (Qi, 2018; Liu, 2019; Xinwei Guo et al., 2020) had a treatment period of >4 weeks. The experimental groups of 16 studies (Mehrbani et al., 2015; Yine and Li, 2017; Yu Zhou et al., 2017; Hu and Feng, 2018; Ma, 2018; Tianming Ma and Liu, 2018; Yang et al., 2018; Zhang, 2018; Hanhua Cao et al., 2019; Li, 2019; QIi, 2019; Qing Wu et al., 2020; Bin Zhao, 2021; Lan, 2021; Liu, 2021; Tianhua Quan et al., 2021) used a combination of CHM and WM treatment, of which four (Yu Zhou et al., 2017; Zhang, 2018; Hanhua Cao et al., 2019; Lan, 2021) used CHM externally, one (Qing Wu et al., 2020) used CHM internally and externally, and 11 (Mehrbani et al., 2015; Yine and Li, 2017; Hu and Feng, 2018; Ma, 2018; Tianming Ma and Liu, 2018; Yang et al., 2018; Li, 2019; QIi, 2019; Bin Zhao, 2021; Liu, 2021; Tianhua Quan et al., 2021) used CHM orally. Among the control groups, one (Mehrbani et al., 2015) study used a placebo, and the remaining 23 used WM (Table 2). Regarding outcomes, all 24 studies reported pruritus indicators using the VAS scores, and eight studies (Wei and Li, 2017; Yine and Li, 2017; Yu Zhou et al., 2017; Liu, 2018; Tianming Ma and Liu, 2018; Liu, 2019; Xinwei Guo et al., 2020; Bin Zhao, 2021) reported DLQI scores. Additionally, 20 studies (Ying Yang et al., 2006; Wei and Li, 2017; Yine and Li, 2017; Yu Zhou et al., 2017; Liu, 2018; Ma, 2018; Qi, 2018; Tianming Ma and Liu, 2018; Yang et al., 2018; Zhang, 2018; Hanhua Cao et al., 2019; Li, 2019; Liu, 2019; Qing Wu et al., 2020; Rui Tao et al., 2020; Yu, 2020; Bin Zhao, 2021; Lan, 2021; Liu, 2021; Tianhua Quan et al., 2021) reported total effective rates, and 13 studies (Ying Yang et al., 2006; Mehrbani et al., 2015; Yine and Li, 2017; Yu Zhou et al., 2017; Hu and Feng, 2018; Qi, 2018; Tianming Ma and Liu, 2018; Liu, 2019; Qing Wu et al., 2020; Rui Tao et al., 2020; Xinwei Guo et al., 2020; Liu, 2021; Tianhua Quan et al., 2021) reported the occurrence of adverse reactions.

### 3.2 Risk of bias assessment

**FIGURE 2:**
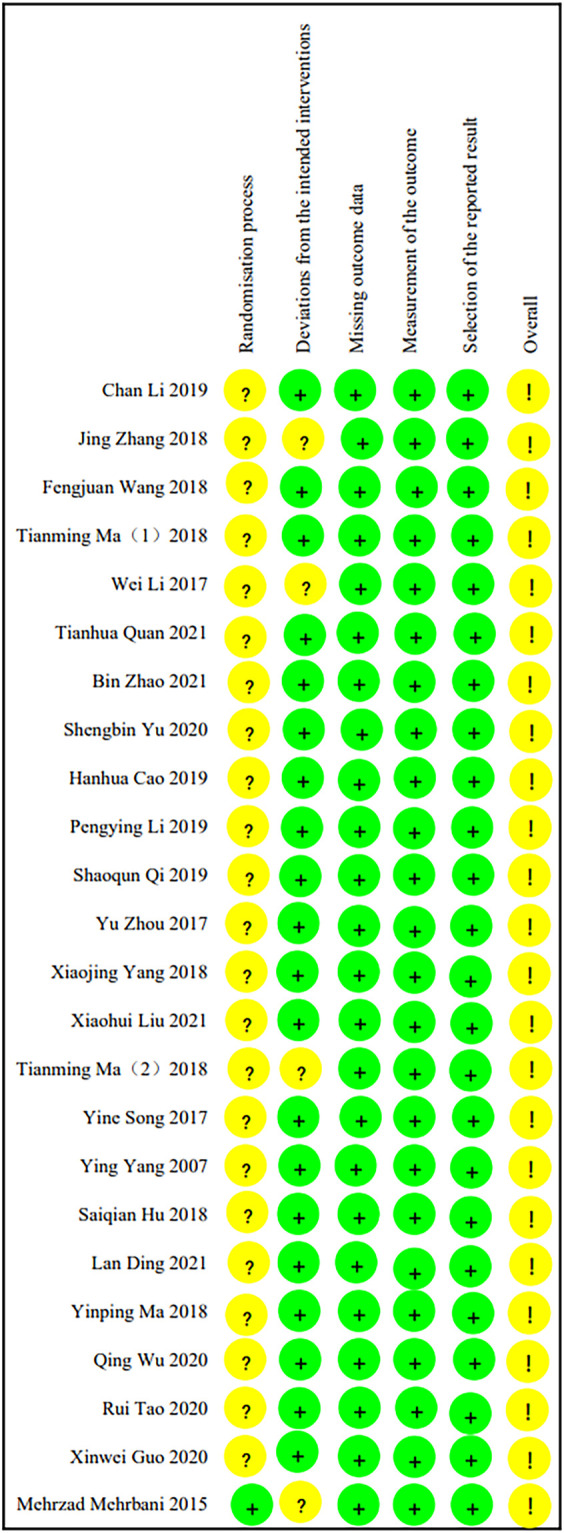
The results for the evaluation of the selected articles using ROB2.0.

**FIGURE 3:**
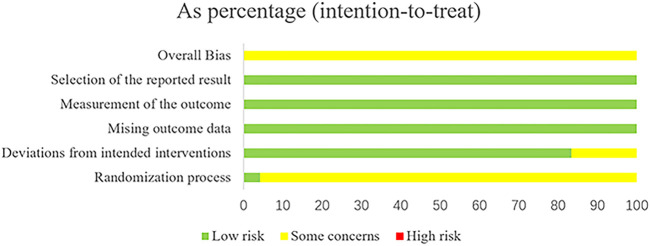
The results for the evaluation of the selected articles using ROB2.0.

The risk of article bias is presented in Figures 2, 3. Only one article (Mehrbani et al., 2015) explicitly mentioned the use of blindness. As other studies did not report participant/person blind or outcome measurement, both performance bias and detection bias in other studies were judged as unclear. In 23 studies (Ying Yang et al., 2006; Wei and Li, 2017; Yine and Li, 2017; Yu Zhou et al., 2017; Hu and Feng, 2018; Liu, 2018; Ma, 2018; Qi, 2018; Tianming Ma and Liu, 2018; Yang et al., 2018; Zhang, 2018; Hanhua Cao et al., 2019; Li, 2019; Liu, 2019; QIi, 2019; Qing Wu et al., 2020; Rui Tao et al., 2020; Xinwei Guo et al., 2020; Yu, 2020; Bin Zhao, 2021; Lan, 2021; Liu, 2021; Tianhua Quan et al., 2021), the risk of bias was considered medium due to the lack of explicit reference to allocation concealment. In four studies (Mehrbani et al., 2015; Wei and Li, 2017; Tianming Ma and Liu, 2018; Zhang, 2018), subjects dropped out of the study. The authors explained the reasons for the dropout, but they still considered a potential risk of bias. The judgment of unclear risk of bias was given to all studies for reporting bias, as none of the studies had study protocols and did not provide sufficient information for further assessment. Information such as the source of funding, sample size calculation, and trial registration was also insufficient to assess other potential biases in the included studies.

### 3.3 Primary outcomes

#### 3.3.1 Pruritus

##### 3.3.1.1 Oral CHM versus placebo

**FIGURE 4:**
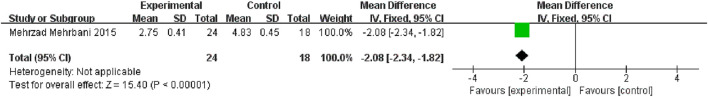
The results of the meta-analysis for the effect of traditional Chinese medicine and placebo on pruritus.

Only one study (Mehrbani et al., 2015) compared CHM with placebo; the CHM and placebo groups had a significant difference in pruritus, as determined by a fixed-effects model (*n* = 42, MD: −2.08; 95% CI = −2.34 to −1.82; *p* < .00001; Figure 4).

##### 3.3.1.2 CHM versus WM

###### 3.3.1.2.1 Oral CHM treatment

**FIGURE 5:**
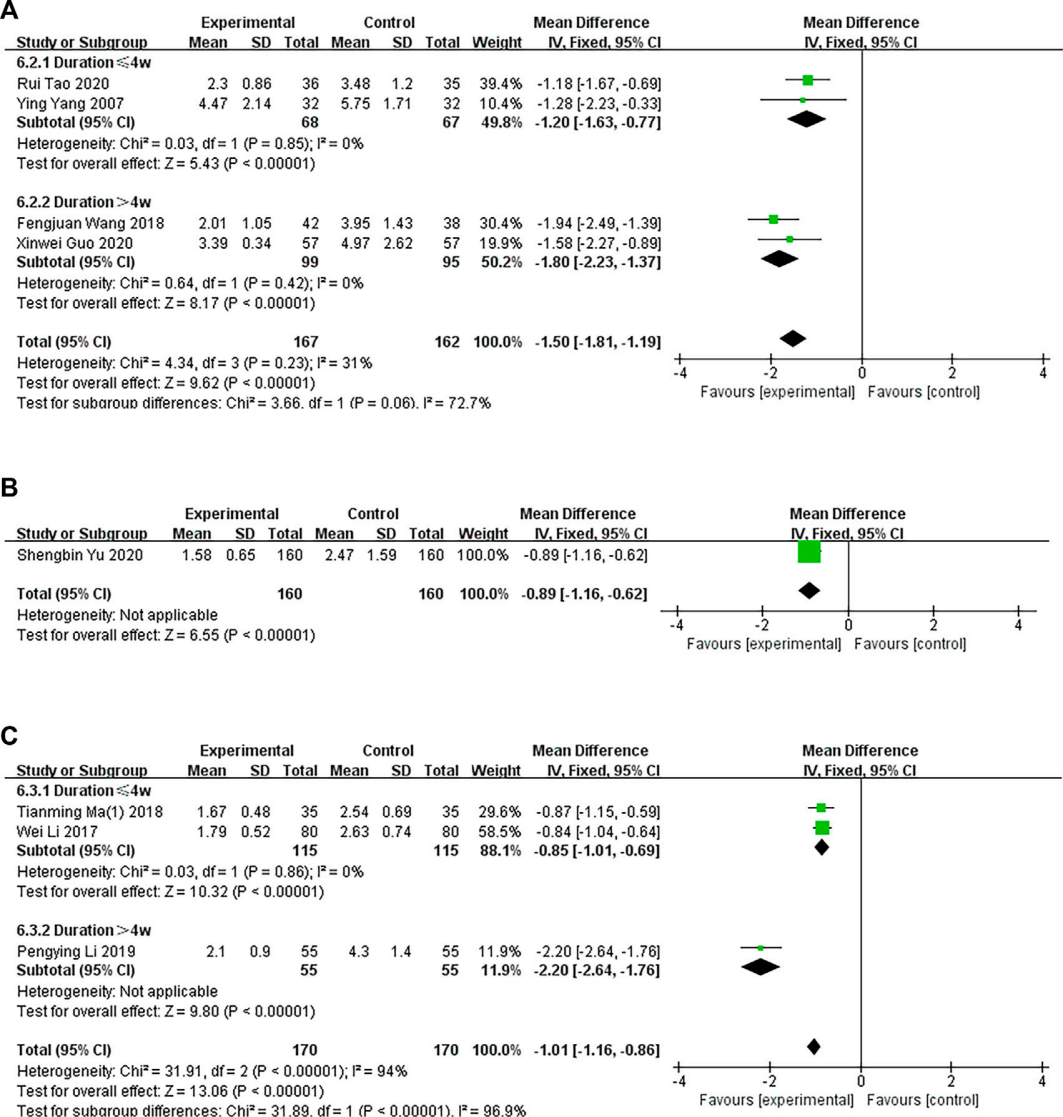
The results of the meta-analysis for the effect of traditional Chinese medicine versus Western medicine on pruritus. **(A)** Oral CHM treatment; **(B)** External treatment with CHM; **(C)** Oral and external treatment with CHM.

In four trials, the experimental groups were administered oral CHM, and the control groups were administered WM. A subgroup analysis of treatment duration showed that two studies (Ying Yang et al., 2006; Rui Tao et al., 2020) had treatment durations <4 weeks (*p* = .85, I^2^ = 0%) and two studies (Qi, 2018; Xinwei Guo et al., 2020) had treatment durations >4 weeks (*p* = .42, I^2^ = 0%). The TCM and WM groups had significant differences in pruritus, as determined by a fixed-effects model (*n* = 135, MD = −1.20, 95% CI = −1.63 to −.77, *p* < .00001; *n* = 194, MD = −1.80, 95% CI = −2.23 to −1.37, *p* < .00001; Figure 5A).

###### 3.3.1.2.2 External treatment with CHM

In one trial (Yu, 2020), the experimental group was administered CHM externally, and the control group was administered WM. The TCM and WM groups had significant differences in pruritus, as determined by a fixed-effects model (*n* = 320, MD = −.89 95% CI = −1.16 to −.62, *p* < .00001; Figure 5B).

###### 3.3.1.2.3 Oral and external treatment with CHM

Three trials compared oral and external CHM treatment with WM. A subgroup analysis of treatment duration showed that two studies (Wei and Li, 2017; Liu, 2018) had treatment durations <4 weeks (*p* = .87, I^2^ = 0%) and one study (Liu, 2019) had treatment duration >4 weeks (*p* < .00001). A fixed-effects model showed that the TCM and WM groups had a significant difference in pruritus after treatment (*n* = 210, MD = −.85, 95% CI = −1.02 to −.68, *p* < .00001; *n* = 110, MD = −2.20, 95% CI = −2.26 to −1.76, *p* < .00001; Figure 5C).

##### 3.3.1.3 Combination of CHM and WM versus WM alone

###### 3.3.1.3.1 Oral CHM treatment

**FIGURE 6:**
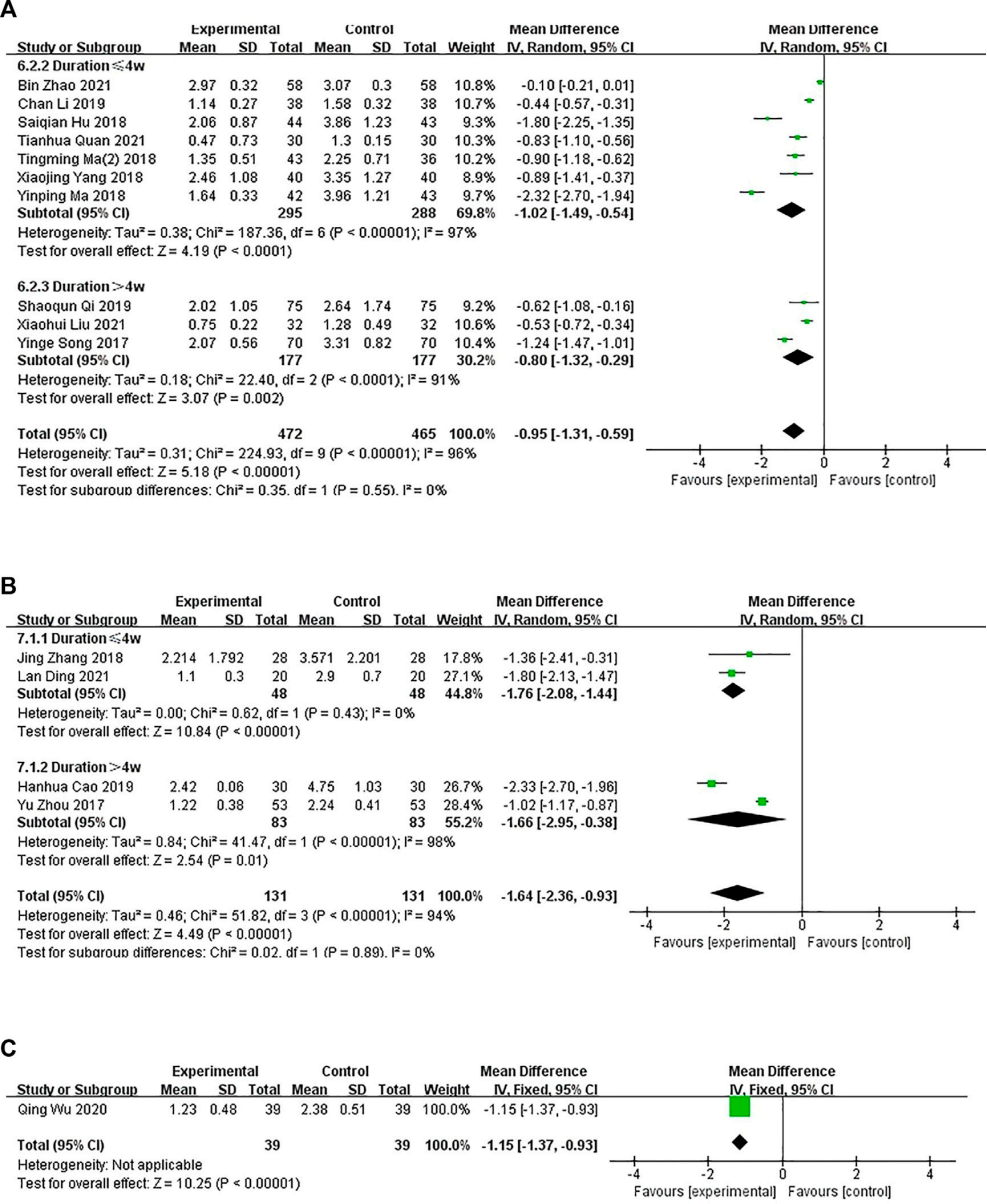
The results of the meta-analysis for the effect of the combination of Chinese herbal medicine and Western medicine versus Western medicine on pruritus. **(A)** Oral CHM treatment; **(B)** External treatment with CHM; **(C)** Oral and external treatment with CHM.

In 11 studies, the experimental group was treated with oral CHM and WM, and the control group was treated with WM. A subgroup analysis of treatment duration showed that the treatment duration was <4 weeks in seven studies (Hu and Feng, 2018; Ma, 2018; Tianming Ma and Liu, 2018; Yang et al., 2018; Li, 2019; Bin Zhao, 2021; Tianhua Quan et al., 2021) (*p* < .0001, I^2^ = 97%) and >4 weeks in three studies (Yine and Li, 2017; QIi, 2019; Liu, 2021) (*p* < .0001, I^2^ = 91%), suggesting some heterogeneity. A random-effects model showed a significant difference between the groups (*n* = 583, MD = −1.02, 95% CI = −1.49 to −.54, *p* < .0001; *n* = 354, MD = −.80, 95% CI = −1.32 to −.29, *p* = .002; Figure 6A). The funnel plot was asymmetrical, indicating publication bias (Supplementary Figure S1).

###### 3.3.1.3.2 External treatment with CHM

In four studies, the intervention group received external treatment with CHM and WM, whereas, the control group received WM treatment only. A subgroup analysis of treatment duration showed that the treatment duration was <4 weeks in two studies (Zhang, 2018; Lan, 2021) (*p* = .43, I^2^ = 0%) and >4 weeks in two studies (Yu Zhou et al., 2017; Hanhua Cao et al., 2019) (*p* < .00001, I^2^ = 98%), suggesting some heterogeneity. A random-effects model showed a significant difference between the groups (*n* = 96, MD = −1.76, 95% CI = −2.08 to −1.44, *p* < .00001; *n* = 166, MD = −1.66, 95% CI = −2.95 to −.38, *p* = .01; Figure 6B). However, the combined analysis showed significant statistical heterogeneity (Chi-squared = 51.82; degrees of freedom = 3; I^2^ = 94%). The cause of heterogeneity was difficult to analyze due to the small number of studies. This might be because the scoring process is subjective.

###### 3.3.1.3.3 Oral and external treatment with CHM

In one study (Qing Wu et al., 2020), the experimental group was administered oral and external treatment with CHM and WM and the control group was administered WM. A fixed-effects model showed significant difference in pruritus between the groups after treatment (*n* = 78, MD = −1.15, 95% CI = −1.37 to −.93, *p* < .0000; Figure 6C).

### 3.4 Secondary outcomes

#### 3.4.1 Dermatology Life Quality Index

##### 3.4.1.1 CHM versus WM

**FIGURE 7:**
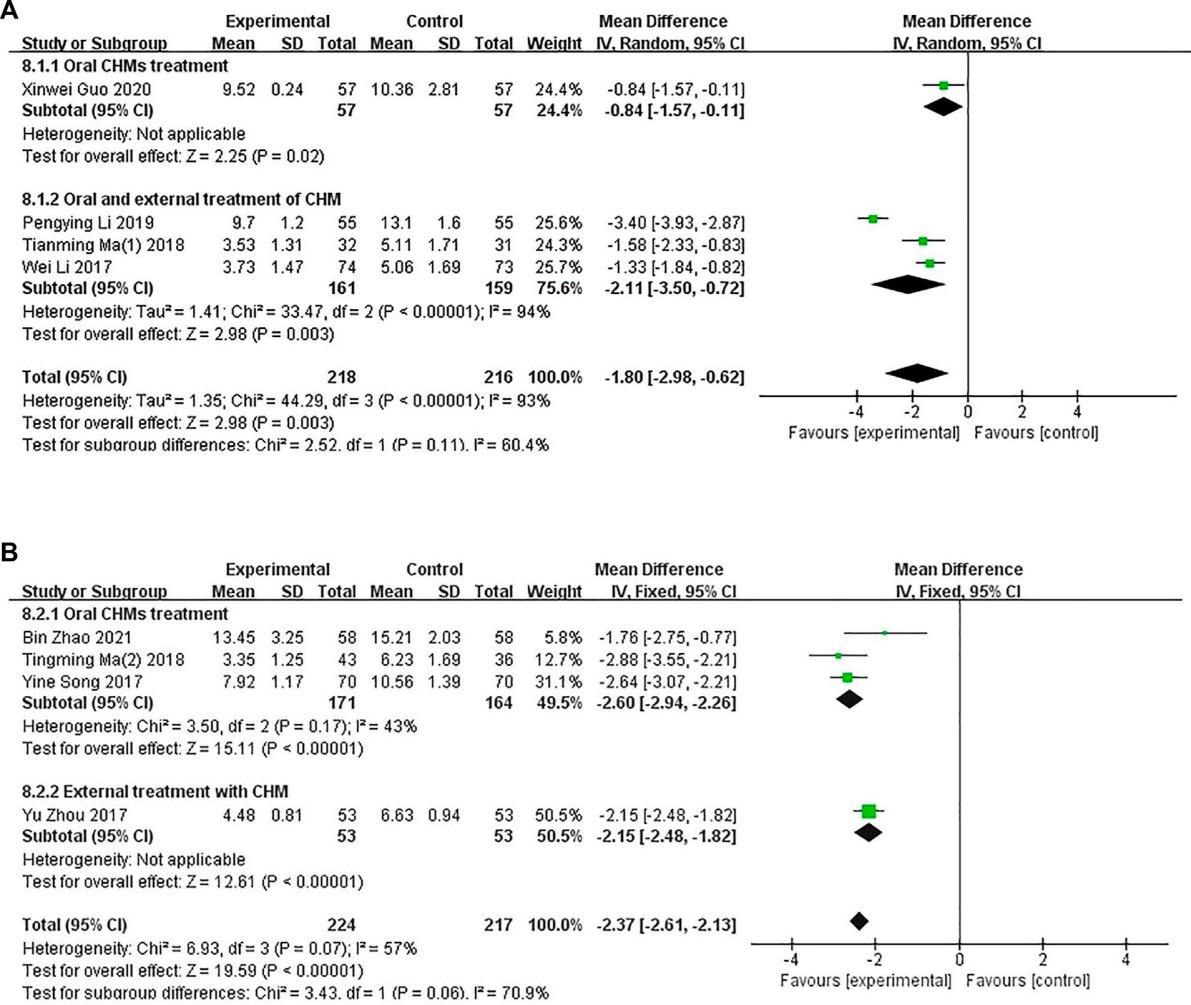
The results of the meta-analysis for the effect of Chinese herbal medicine or a combination of CHM and Western medicine (WM) versus WM on the Dermatology Life Quality Index score. **(A)** CHM versus WM; **(B)** Combination of CHM and WM versus WM.

In four studies involving 434 patients, the DLQI was applied to assess the quality of life of patients with CP. A subgroup analysis showed that one (Xinwei Guo et al., 2020) study compared the administration of oral CHM with WM (*p* = .02), and three studies (Wei and Li, 2017; Liu, 2018; Liu, 2019) compared the administration of oral and external CHM treatment with WM (*p* < .00001, I^2^ = 94%). The pooled analysis showed that the DLQI scores of patients in the CHM groups were lower than those of patients in the control groups (MD = −1.80, 95% CI = −2.98 to −.62, *p* = .003 < .05; Figure 7A).

##### 3.4.1.2 Combination of CHM and WM versus WM

A combination of CHM and WM was used in four studies (Figure 7B). A subgroup analysis was conducted to determine the differences in the methods of administration. Three studies (Yine and Li, 2017; Tianming Ma and Liu, 2018; Bin Zhao, 2021) compared the administration of oral CHM with WM (*p* = .17, I^2^ = 43%), and one study (Yu Zhou et al., 2017) compared the administration of oral and external CHM treatment with WM treatment (*p* < .00001). The pooled analysis showed that the DLQI scores of patients in the CHM and WM groups were significantly lower than those of patients in the control groups (MD = −2.37, 95% CI = −2.61 to −2.13, *p* < .00001).

#### 3.4.2 Effective rate

In total, 20 studies (Ying Yang et al., 2006; Wei and Li, 2017; Yine and Li, 2017; Yu Zhou et al., 2017; Liu, 2018; Ma, 2018; Qi, 2018; Tianming Ma and Liu, 2018; Yang et al., 2018; Zhang, 2018; Hanhua Cao et al., 2019; Li, 2019; Liu, 2019; Qing Wu et al., 2020; Rui Tao et al., 2020; Yu, 2020; Bin Zhao, 2021; Lan, 2021; Liu, 2021; Tianhua Quan et al., 2021) consisting of 1,895 patients reported the effective rate. Among them, seven studies compared CHM with WM (Ying Yang et al., 2006; Wei and Li, 2017; Liu, 2018; Qi, 2018; Liu, 2019; Rui Tao et al., 2020; Yu, 2020), and 13 studies compared a combination of CHM and WM with the same WM (Yine and Li, 2017; Yu Zhou et al., 2017; Ma, 2018; Tianming Ma and Liu, 2018; Yang et al., 2018; Zhang, 2018; Hanhua Cao et al., 2019; Li, 2019; Qing Wu et al., 2020; Bin Zhao, 2021; Lan, 2021; Liu, 2021; Tianhua Quan et al., 2021).

##### 3.4.2.1 CHM versus WM

**FIGURE 8:**
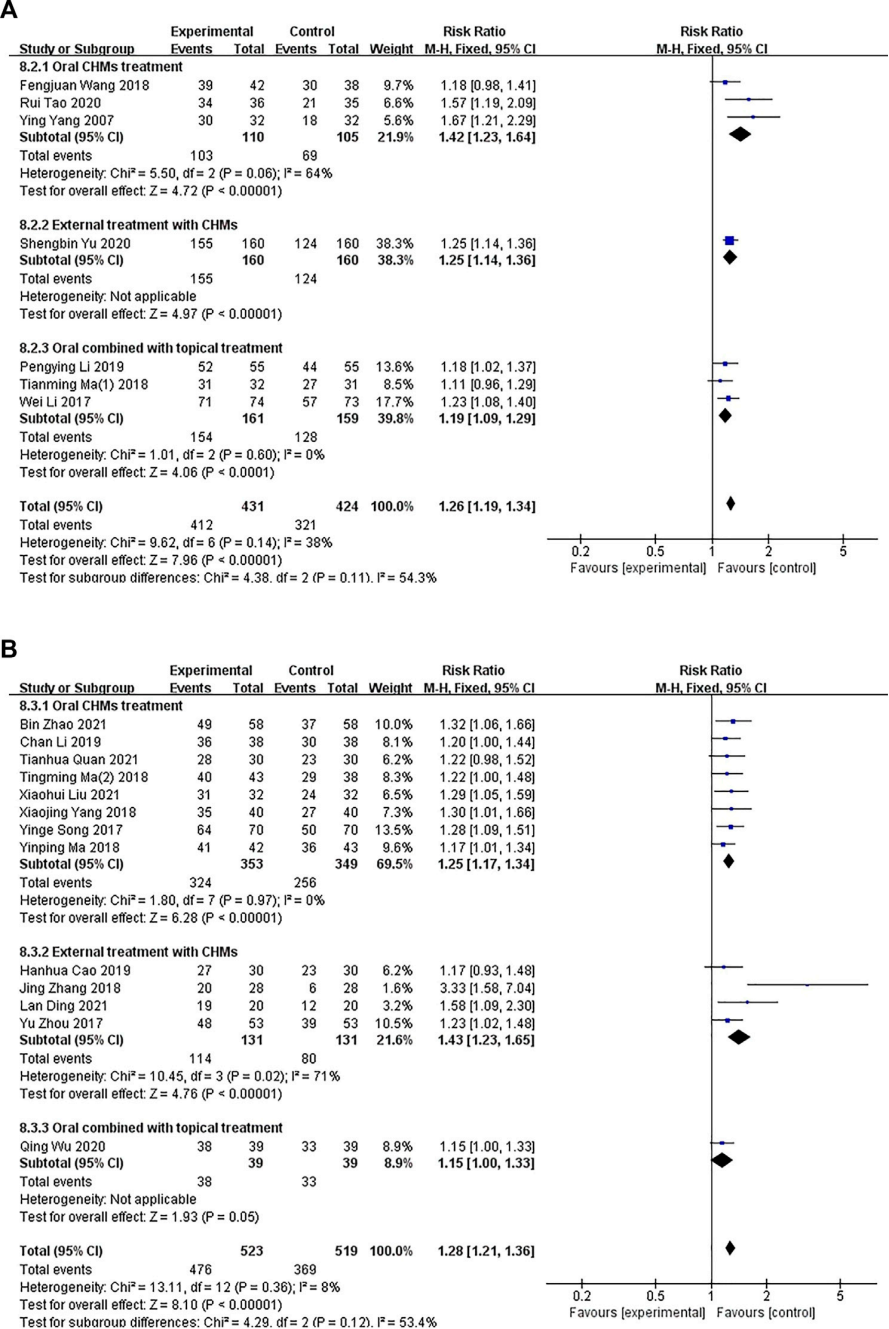
The results of the meta-analysis for the effect of Chinese herbal medicine (CHM) or a combination of CHM and Western medicine (WM) versus WM on the effective rate. **(A)** CHM versus WM; **(B)** CHM versus WM.

Seven studies compared CHM with WM (*p* = .14, I^2^ = 38%), and a fixed-effects model was used to perform the meta-analysis. A subgroup analysis was conducted to determine the differences in the methods of administration. Three studies (Ying Yang et al., 2006; Qi, 2018; Rui Tao et al., 2020) compared the administration of oral CHM with WM (I^2^ = 64%, RR: 1.42; 95% CI = 1.23–1.64, *p* < .00001), one study (Yu, 2020) compared the administration of external CHM with WM (RR: 1.25; 95% CI = 1.14–1.36), *p* < .00001), and three studies (Wei and Li, 2017; Liu, 2018; Liu, 2019) compared the administration of oral and external CHM treatment with WM (I^2^ = 0%, RR: 1.19; 95% CI = 1.09–1.29; *p* < .0001). The results of the meta-analysis showed that the effective rate of patients in the CHM groups was higher than those of patients in the control groups (Figure 8A).

##### 3.4.2.2 Combination of CHM and WM versus WM

In total, 13 studies compared a combination of CHM and WM with WM (*p* = .36, I^2^ = 8%). A fixed-effects model was used for conducting the meta-analysis. A subgroup analysis was conducted to examine the differences in the administration methods used. Eight studies (Yine and Li, 2017; Ma, 2018; Tianming Ma and Liu, 2018; Yang et al., 2018; Li, 2019; Bin Zhao, 2021; Liu, 2021; Tianhua Quan et al., 2021) compared the administration of oral CHM and WM with WM (I^2^ = 0%, RR: 1.25; 95% CI = 1.17–1.34, *p* < .00001), four studies (Yu Zhou et al., 2017; Zhang, 2018; Hanhua Cao et al., 2019; Lan, 2021) compared the administration of external CHM and WM with WM (I^2^ = 71%, RR: 1.43; 95% CI = 1.23–1.65, *p* < .00001), and one study (Qing Wu et al., 2020) compared the administration of oral and external CHM and WM with WM (RR: 1.15; 95% CI = 1.00–1.33; *p* = .05). The results of the meta-analysis showed a significant difference in the effective rate between the groups when the administration method of CHM was oral or external (*p* < .00001), but no significant difference was observed between the groups when a combination of oral and external methods of CHM administration was performed (*p* = .05; Figure 8B).

#### 3.4.3 Recurrence rate

Four studies (Qing Wu et al., 2020; Rui Tao et al., 2020; Bin Zhao, 2021; Tianhua Quan et al., 2021) consisting of 185 patients reported the recurrence rate. Among them, one study (Rui Tao et al., 2020) compared CHM with WM, where the patients were followed up for 3 months after treatment. Three studies compared a combination of CHM and WM with the same WM, in which, two studies (Bin Zhao, 2021; Tianhua Quan et al., 2021) had a follow-up for 1 month after treatment, and one study (Qing Wu et al., 2020) had a follow-up for 3 months after treatment.

##### 3.4.3.1 CHM versus WM

**FIGURE 9:**
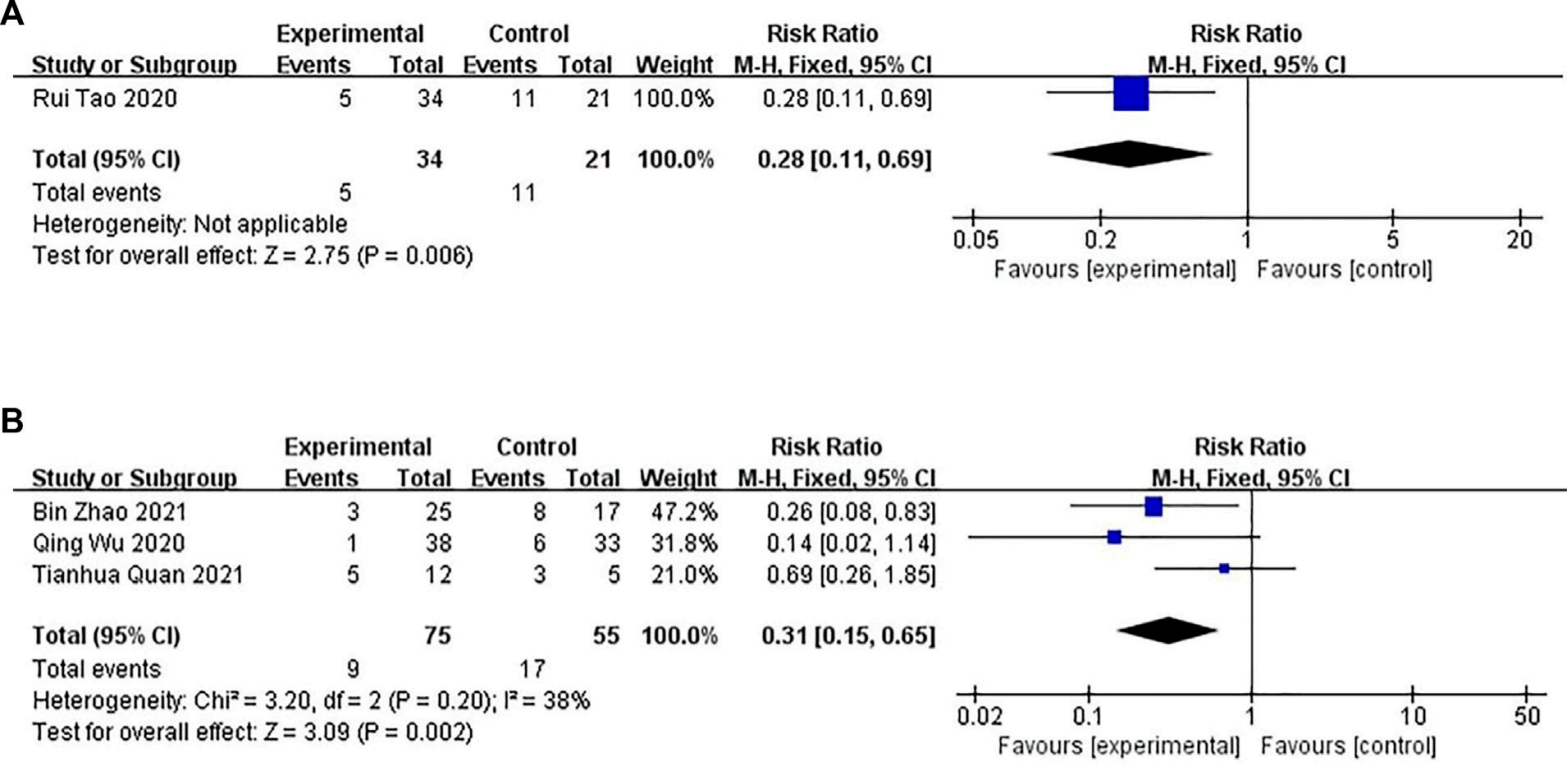
The results of the meta-analysis for the effect of Chinese herbal medicine (CHM) or a combination of CHM and Western medicine (WM) versus WM on the recurrence rate. **(A)** CHM versus WM; **(B)** Combination of CHM and WM versus WM.

A fixed-effects model was used for conducting the meta-analysis of the studies that compared CHM with WM. Significant differences were observed in the recurrence rate between the CHM and WM groups (*n* = 55, RR: .28; 95% CI = .11–.69; *p* = .006 < .01; Figure 9A).

##### 3.4.3.2 Combination of CHM and WM versus WM

Three studies compared the combination of CHM and WM versus WM (*p* = .20, I^2^ = 38%). A fixed-effects model was used for conducting the meta-analysis. The recurrence rate between the combination of CHM and WM groups differed significantly (*n* = 130, RR: .31; 95% CI = .15–.65; *p* = .002 < .01; Figure 9B).

#### 3.4.4 Safety

In total, 13 studies conducted with 1,094 patients reported adverse events (AEs). One study compared CHM with a placebo, and the AEs of the CHM group included anorexia and gastrointestinal discomfort (such as indigestion). Five studies compared the combination of CHM with WM. AEs in the CHM groups included dryness of the mouth, insomnia, nausea, headache, dizziness, and gastrointestinal side effects (such as nausea, vomiting, constipation, stomach discomfort, and loss of appetite). Seven studies compared the combination of CHM and WM with the same WM and reported AEs, including rash, fatigue, insomnia, gastrointestinal side effects, headache, and dizziness. No serious adverse reactions were observed.

**FIGURE 10:**
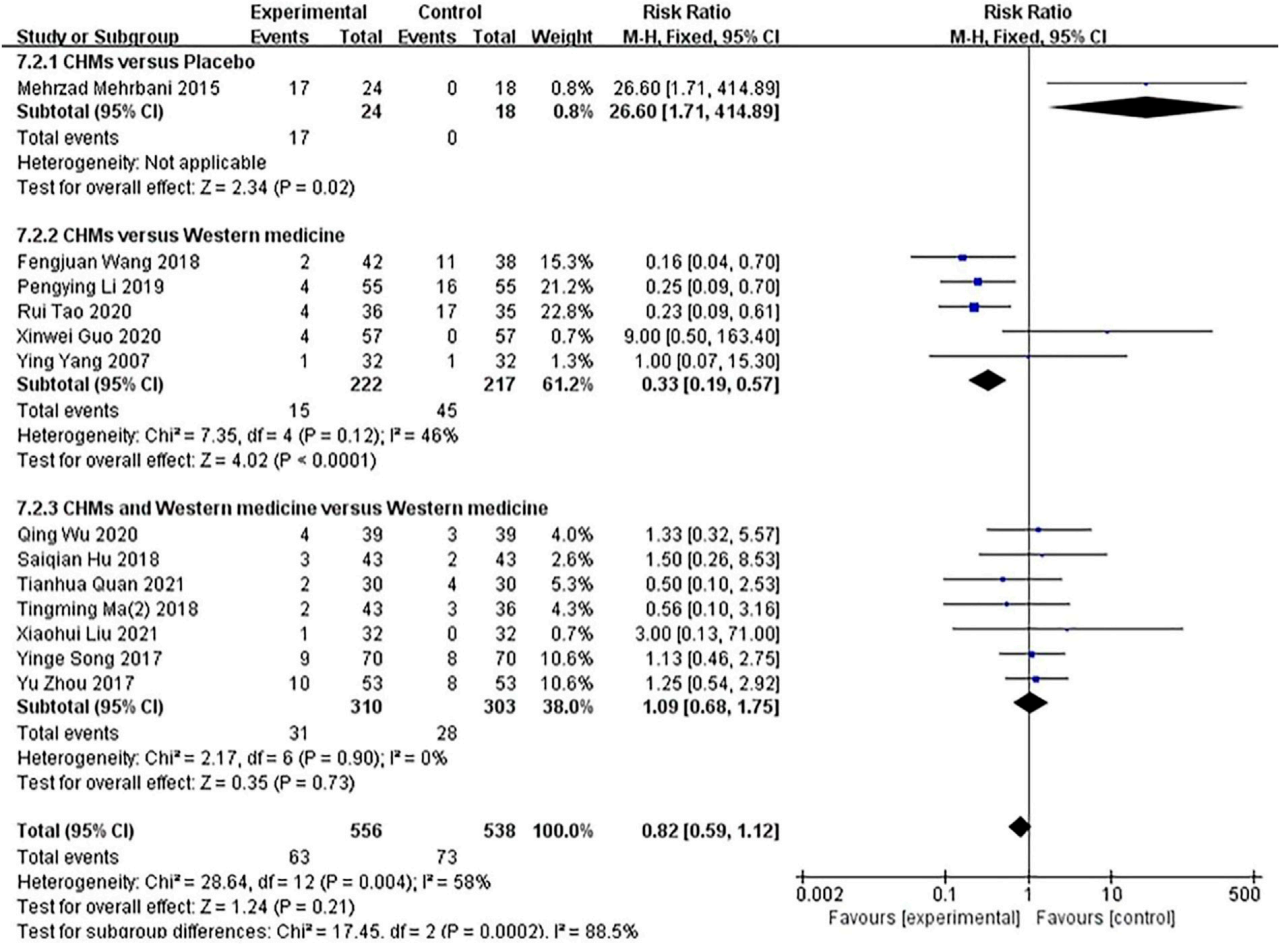
The results of the meta-analysis for the effect of Chinese herbal medicine (CHM) or a combination of CHM and Western medicine (WM) versus WM on the rate of adverse effects.

A subgroup analysis of treatment methods showed that in one study, the incidence of adverse reactions was reported in the CHM group compared with the placebo group (*p* = .02), and a fixed-effects model showed no significant difference between the groups (*n* = 42, RR = 26.60, 95% CI = 1.71–414.89, *p* = .02). Five studies compared CHM with WM (*p* = .12, I^2^ = 46%), and a fixed-effects model showed that the rate of adverse effects on patients in the CHM groups was higher than that on the participants in the control groups (*n* = 439 RR = .33, 95% CI = .19–.57, *p* < 0,001). Seven studies compared the combination of CHM and WM with WM (*p* = .90, I^2^ = 0%), and a fixed-effects model showed that the incidence of adverse reactions between the groups was not significantly different (*n* = 613, RR = 1.09, 95% CI = .68–1.75, *p* = .73; Figure 10).

### 3.5 GRADE for the main comparisons

The GRADE quality of evidence was evaluated for each outcome. The quality of evidence for pruritus, the DLQI score, and the recurrence rate were low or very low; the quality of evidence for the effective rate was high, and the quality of evidence for the rate of adverse effects was moderate (Table 4).

**TABLE 4:** Summary of GRADE.

Quality assessment							No of patients		Effect		Quality	Importance
No of studies	Design	Risk of bias	Inconsistency	Indirectness	Imprecision	Reporting bias	Intervention	Control	Relative (95% CI)	Absolute
Pruritus (24 studies)	RCT	serious^1^	Serious^2^	no serious indirectness	no serious imprecision	reporting bias^4^	1,165	1,147	—	MD 1.01 lower (1.06–.95 lower)	⊕ΟΟΟ VERY LOW^1,2,4^	IMPORTANT
DLQI (8 studies)	RCT	Serious^1^	very serious^2^	no serious indirectness	no serious imprecision	undetected	451	444	—	MD 2.2 lower (2.38–2.01 lower)	⊕⊕ΟΟ LOW^1,2^	IMPORTANT
Effective rate (17 studies)	RCT	serious^1^	no serious inconsistency	no serious indirectness	no serious imprecision	none	768/826 (93%)	597/814 (73.3%)	RR 1.27 (1.21–1.32)	198 more per 1,000 (from 154 more to 235 more)	⊕⊕⊕⊕ HIGH^1^	IMPORTANT
								76.7%		207 more per 1,000 (from 161 more to 245 more)		
Recurrence rate (4 studies)	RCT	serious^1^	no serious inconsistency	no serious indirectness	Serious^3^	undetected	14/109 (12.8%)	28/76 (36.8%)	RR .3 (.17–.53)	258 fewer per 1,000 (from 173 fewer to 306 fewer)	⊕⊕ΟΟ LOW^1,3^	IMPORTANT
								49.7%		348 fewer per 1,000 (from 234 fewer to 413 fewer)		
Adverse effect rate	RCT	serious^1^	no serious inconsistency	no serious indirectness	no serious imprecision	none	63/556 (11.3%)	73/538 (13.6%)	RR .82 (.59–1.12)	24 fewer per 1,000 (from 56 fewer to 16 more)	⊕⊕⊕Ο MODERATE^1^	IMPORTANT
								8.3%		15 fewer per 1,000 (from 34 fewer to 10 more)		

1. Lacking blinding and randomization and allocation are unclear; 2. Substantial heterogeneity; 3. Small sample size; 4. The outcomes of Egger’s test suggested publication bias.

### 3.6 Description of the CHM

Several herbs were included in the 24 studies evaluated. The top 15 most frequently used herbs were used more than six times and included Divaricate Saposhniovia Root, Liquorice root, Light yellow Sophora Root, Chinese Angelica, Paeonia lactiflora, Densefruit Pittany Root-bark, Wolfiporia cocos, Fine leaf Schizonepeta Herb, Szechuan Lovage Rhizome, Rehmannia Glutinosa, Fructus Kochiae, Common Cnidium Fruit, Cicada Slough, Large head Atractylodes Rh, and Dan-Shen root (Table 5).

**TABLE 5:** The 15 most frequently used ingredients of Chinese herbal medicine in 24 prescriptions.

Accepted name	Chinese name	Family	Number of studies (%)
*Saposhnikovia divaricata* (Turcz. ex Ledeb.) Schischk	Fangfeng	*Apiaceae*	16 (66.67)
*Glycyrrhiza glabra* L	Gancao	*Fabaceae*	15 (62.5)
*Sophora flavescens* Aiton	Kushen	*Fabaceae*	12 (50)
*Angelica sinensis* (Oliv.) Diels	Danggui	*Apiaceae*	12 (50)
*Paeonia lactiflora* Pall	Shaoyao	*Paeoniaceae*	11 (45.83)
*Dictamnus dasycarpus* Turcz	Baixianpi	*Rutaceae*	10 (41.67)
*Smilax glabra* Roxb	Fuling	*Smilacaceae*	10 (41.67)
*Sesamum indicum* L	Jingjie	*Pedaliaceae*	10 (41.67)
*Conioselinum anthriscoides* “Chuanxiong”	Chuanxiong	*Apiaceae*	9 (37.50)
*Rehmannia glutinosa* (Gaertn.) DC	Dihuang	*Orobanchaceae*	8 (33.33)
*Bassia scoparia* (L.) A. J. Scott	Difuzi	*Amaranthaceae*	79 (29.17)
*Cnidium monnieri* (L.) Cusson	Shechuangzi	*Apiaceae*	7 (29.17)
*Tabernaemontana divaricata* (L.) R.Br. ex Roem. and Schult	Chantui	*Apocynaceae*	6 (25)
*Atractylodes macrocephala* Koidz	Baizhu	*Asteraceae*	6 (25)
*Salvia miltiorrhiza* Bunge	Danshen	*Lamiaceae*	6 (25)

## 4 Discussion

### 4.1 Summary of evidence

Many studies have shown that CHM is effective in treating CP; however, no available treatment method meets the requirements of evidence-based medicine. Systematic reviews and meta-analyses contribute to the development of evidence-based medicine and are the best source of clinical evidence [15]. We conducted a systematic review of RCTs on CHM for CP. In this systematic review, we evaluated the efficacy and safety of CHM (or a combination of CHM and WM) in treating CP. We included 24 RCTs, with 2,288 patients. The results showed that CHM (or a combination of CHM and WM) was significantly more effective than WM in improving pruritus degree, the DLQI score, the effective rate, and the recurrence rate of CP.

We found that the treatment of CP by administering CHM significantly improved the degree of pruritus compared to the placebo. CHM had a more significant effect than WM on the improvement of the degree of pruritus, the DLQI score, and the effective rate in CP. Subgroup analysis was conducted on the groups of participants who were treated *via* different drug administration methods and treatment courses. The results showed that oral administration was the most effective, followed by a combination of oral and external treatment. The effect of the treatment on reducing itching improved with the increase in the duration of treatment. These results showed that performing monotherapy with CHM can improve the symptoms of CP patients. Regarding the comparison of the combination of CHM and WM with WM, the meta-analysis showed that CHM combined with WM in CP treatment is significantly more effective in improving the degree of pruritus, the DLQI score, the effective rate, and the recurrence rate. The combination of CHM and WM was the most effective when topically applied. These results indicated that CHM as an adjunctive therapy can improve the symptoms of CP patients.

Regarding the effect of the patient recurrence rate, the disease recurrence rate in the CHM and combination of CHM and WM treatment groups was lower than that in the WM treatment group. Thirteen studies that investigated the safety of CHM treatment reported AEs, including dryness of the mouth, insomnia, nausea, dizziness, rash, fatigue, gastrointestinal side effects, headache, and dizziness. The results of the meta-analysis for the effect of the AEs showed no significant difference in the incidence of AEs between the CHM and placebo groups, between the CHM and WM groups, and between the combination of CHM and WM and WM-only groups. This indicated that CHM might be recommended for treating patients with CP.

### 4.2 Implications for practice

This meta-analysis revealed that CHM is safe and might be used for monotherapy or adjuvant therapy to improve pruritus and the DLQI score of patients with CP. A descriptive analysis based on included RCTs indicated a great diversity in the detailed composition of the herbs in the CHM prescribed for patients with CP. We found that among the CHM included in the studies, Saposhnikovia divaricata (Turcz. ex Ledeb.) Schischk, *Glycyrrhiza glabra* L, *Sophora flavescens* Aiton, *Angelica sinensis* (Oliv.) Diels, *Paeonia lactiflora* Pall, *Dictamnus dasycarpus* Turcz, *Smilax glabra* Roxb, *Sesamum indicum* L, *Conioselinum anthriscoides* “Chuanxiong,” *Rehmannia glutinosa* (Gaertn.) DC, *Bassia scoparia* L.) A.J. Scott, *Cnidium monnieri* L.) Cusson, *Tabernaemontana divaricata* L.) R. Br. ex Roem. and Schult, *Atractylodes macrocephala* Koidz, *Salvia miltiorrhiza* Bunge were frequently used as the most effective prescriptions for treating chronic itching. These findings should be further considered when formulating commonly used CHM for CP treatment.

### 4.3 Limitations

This study had several limitations: (Rajagopalan et al., 2017) The number and sample size of the included studies were relatively small, which affected the reliability of the conclusions (Villa-Arango et al., 2019) Among the included studies, 11 studies did not report allocation concealment, only one study conducted blinded trials, and the trials in the other studies were not blinded; thus, a certain risk of bias existed (Ständer et al., 2007) The evaluation standard of the degree of itching was single and highly subjective, and the risk of deviation was high (Weisshaar et al., 2019) The formulation composition, dosage, administration method, and CHM treatment duration in RCTs varied widely across studies. Clinical heterogeneity compromised the validity of our findings. The publication bias caused by all the studies being published in China might have partly exaggerated the efficacy of CHM.

### 4.4 Implications for research

Using CHM for monotherapy or adjuvant therapy is effective in treating CP, and this review provides existing evidence that might help to shape the design of future trials. Although double‒blinded trials may be difficult due to the nature of CHM treatment, study investigators should consider alternative strategies to minimize the risk of performance bias. The trials could have also at least blinded the individuals who assessed the trial outcomes. After incorporating these methodologic precautions, study investigators should acknowledge the potential biases arising from the lack of blinding, and address them appropriately in the limitations of their study. For example, the use of the CONSORT 2017 Chinese Herbal Prescription Expansion (Cheng et al., 2017) to report the results of RCTs involving herbal interventions, the use of the CONSORT 2010 Statement (Piaggio et al., 2012), and the RCT design protocol used to study CHM [18] to establish and report RCTs for CHM treatment. Although the findings of this systematic review suggested that the use of CHM treatment might be relatively safe in patients with CP, further studies are needed to confirm our findings. Bian et al. (Bian et al., 2010) established a standard format for reporting adverse drug reactions in CHM, which might improve the ways to report adverse drug reactions. Regardless, both study investigators and authors should ensure a strict methodology and proper reporting, to reduce potential biases in trials evaluating the effectiveness of herbal medicine for the treatment of CP. Additionally, the effectiveness of TCM depends on accurately differentiating and treating the syndrome. Therefore, drug prescriptions must be distinguished based on different syndromes of diseases. When evaluating the therapeutic effects of CHM treatment, syndrome differentiation and treatment should be accurately conducted, and individualized TCM prescriptions should be formulated to treat specific diseases. For example, a study (Bensoussan et al., 1998) showed that the administration of personalized CHM for treating irritable bowel syndrome had better effects than a generic hypnotic prescription. Therefore, appropriate drugs prepared from common herbs should be administered in clinical practice based on specific disease syndromes to improve the efficacy of CHM in treating CP.

## 5 Conclusion

This meta-analysis and systematic review of 24 RCTs consisted of 2,288 patients. Low certainty evidence suggested that CHM used with or without WM, compared with WM, might have significantly better effects on alleviating pruritus, increasing DLQI scores, and improving the effective rate in patients with CP in clinical practice. Administering CHM did not increase the risk of adverse events. However, more high-quality RCTs are needed to confirm the effectiveness and adverse events of CHM in the treatment of CP.

## Data Availability

The original contributions presented in the study are included in the article/Supplementary Material, further inquiries can be directed to the corresponding author.

## Abbreviations

CP, chronic pruritus; RCT, randomized controlled trial; RR, risk ratio; MD, mean difference; VAS, visual analogue scale; CHM, Chinese herbal medicine; WM, conventional western medicine treatment; DLQI, Dermatology Life Quality Index; AE, adverse event; AD, atopic dermatitis; GRADE, Grading of Recommendations Assessment; Development, and Evaluation; CI, confidence interval; TCM, traditional Chinese medicine.
