# Supplementary material for: Efficacy and safety of Chinese herbal medicine in the treatment of chronic pruritus: A systematic review and meta-analysis of randomized controlled trials
Source: Front Pharmacol. 2023 Jan 12;13:1029949. doi: 10.3389/fphar.2022.1029949 (PMC9877228; doi:10.3389/fphar.2022.1029949)
Supplement: Supplementary file 1 [file Table1.docx]

# Supplementary Table 1

Supplementary Table 1: The medicinals and dosages used in the original studies

| Study | Formulas | Medicinals and dosages | Source |
| --- | --- | --- | --- |
| Chan Li (2019) | Xiaoyang Decoction | The dried root of *Astragalus mongholicus* Bunge (*huáng qí*)20g, the dried root of *Actaea cimicifuga* L(*shēng má*)20g, the dried velamen of *Dictamnus dasycarpus* Turcz(*bái xiān pí*)15g, *the dried root of Paeonia lactiflora Pall. (bái sháo)*15g, the dried ripe fruit of *Arctium lappa* L（*niú bàng zǐ*）15g, the dried root of *Angelica sinensis* (Oliv.) Diels(*dāng guī*) 10g, the dried root of *Saposhnikovia divaricata* （Turcz.）Schischk（*fáng fēng*）10g, the dry aboveground portion of *Equisetum hyemale* L(*mù zéi*)10g, *the ried rhizome of Cynanchum paniculatum（Bge.）Kitag.（xú cháng qīng）* 10g, the dried root and rhizome of *Glycyrrhiza glabra* L. (*gān căo*), 5g. | Prepared by Chan et al |
| Jing Zhang（2018） | Traditional Chinese medicine bath | The dried root of *Salvia miltiorrhiza* Bge（*dān shēn*） 50g, *the died flowers of Carthamus tinctorius L (hóng huā)* 20g, the ried rhizome of *Rehmannia glutinosa Libosch* （*dì huáng*）50g, the died root bark of *Lycium chinense Mill. or Lycium barbarum* L *(dì gǔ pí)* 50g, the dried root of *Sophora flavescens* Aiton *(kŭ shēn)* 40g, the dried velamen of *Dictamnus dasycarpus* Turcz *(bái xiān pí)* 40g, the dried ripe fruit of *Kochia scoparia* (L.) Schrad (dì fū zǐ) 40g, the dried ripe fruit of *Cnidium monnieri*（L.）Cuss（*shé chuáng zǐ*） 30g, the dried herb of *Scleromitrion diffusum* (Willd.) R.J. Wang (*bái huā shé shé căo*) 30g. | Prepared by Zhang et al |
| Fengjuan Wang（2018） | Jianpi Qushi Decoction | The dried root of *Salvia miltiorrhiza* Bge*（dān shēn）* 20g, the dried rhizome of *Atractylodes macrocephala* Koidz*. (bái zhú)* 15g, the dried sclerotium of *Poria cocos* (Schw.) Wolf *(fú líng)* 10g, the died root bark of *Paeonia suffruticosa* Andr *（mǔ dān pí）* 10g, the dried root of *Saposhnikovia divaricata* （Turcz）Schischk*（fáng fēng）*10g, the dried rhizome of *Alisma orientale*（Sam.）Juzep.or*Alisma plantago-aquatica* Linn (*zé xiè*) 10g, the dried ripe peel of *Citrus × aurantium* L*. (chén pí)* 10g, the dried root of *Paeonia lactiflora* Pall*. (bái sháo)*10g, the died root bark of *Lycium chinense* Mill*. or Lycium barbarum* L *(dì gǔ pí)* 15g, the dried rhizome of *Dioscorea oppositifolia* L. *(shān yào)* 10g, *Orthosiphon aristatus var. aristatus （huá shí）* 10g, the dried velamen of *Dictamnus dasycarpus* Turcz *(bái xiān pí)* 10g, the dried ripe kernel of *Coix lacryma-jobi* L. *(yì yĭ rén)* 10g, the dried root and rhizome of *Glycyrrhiza glabra* L. *(gān căo)* 6g. | Prepared by Wang et al |
| Tianming Ma（1）（2018） | Chinese herbal washing externally and drinking internally | The ried rhizome of *Rehmannia glutinosa* Libosch *（dì huáng）* 20g, the dried root of *Angelica sinensis* (Oliv.) Diels *(dāng guī)*15g, the dried rhizome of *Ophiopogon japonicus* （L. f）Ker-Gawl *（mài dōng）* 20g, the dried root of *Paeonia lactiflora* Pall. *(bái sháo)* 30g, the died root bark of *Paeonia suffruticosa* Andr *（mǔ dān pí）*20g, the dried root of *Sophora flavescens* Aiton *(kŭ shēn)* 20g, the dried ripe fruit of *Tribulus terrestris* L （*jí lí*） 20g, the dried root of *Saposhnikovia divaricata* （Turcz）Schischk*（fáng fēng）*15g, the dried velamen of *Dictamnus dasycarpus* Turcz *(bái xiān pí)* 20g. | Prepared by Ma et al |
| Wei Li（2017） | Qingshi Zhiyang ointment+Modified Xiaofeng Zhiyang Decoction | The dried processed product of *leaf or stem and leaf of Strobilanthes cusia* (Nees) Kuntze *(qīng dài), calamine（lú gān shí）, Orthosiphon aristatus var. aristatus（shí gāo）, Orthosiphon aristatus var. aristatus （huá shí）,* the dried root of *Sophora flavescens* Aiton *(kŭ shēn),* the dried bark of *Phellodendron chinense* C.K. Schneid. *(huáng băi), Cinnamomum camphora (L.) J. Presl （bīng piàn）, Olive oil, vaseline +* the dry body of *Buthus martensii* Karsch *(quán xiē)*3g, *Lavandula angustifolia* Mill（chán yī）10g, the died body of *Zaocys dhumnades* (Cantor) (*wū shāo shé*) 10g, *lufengfang* 10g, the dried velamen of *Dictamnus dasycarpus* Turcz*(bái xiān pí)* 15g, the ried rhizome of *Cynanchum paniculatum*（Bge.）Kitag.*（xú cháng qīng）* 15g, the dried root of *Paeonia lactiflora* Pall. *(bái sháo)* 20g, the dried rhizome of *Conioselinum anthriscoides 'Chuanxiong' (chuān xiōng)* 10g, the dried root of *Salvia miltiorrhiza* Bge（*dān shēn*）20g. | Beijing University of Chinese Medicine EastHospital preparation room, China. |
| Tianhua Quan (2021) | Pingwei Xiaozhen Decoction | The dry aboveground portion of *Schizonepeta tenuifolia* Briq （*jīng jiè*） 15g, the dried root of *Saposhnikovia divaricata* （Turcz）Schischk*（fáng fēng）* 15g, the dried root and rhizome of *Rheum palmatum L.* (*dà huáng*) 6g, *Glauber's salt（máng xiāo）* 9g, the dried rhizome of *Conioselinum anthriscoides 'Chuanxiong' (chuān xiōng)* 10g, the dried root of *Angelica sinensis* (Oliv.) Diels (*dāng guī*) 20g, the dried root of *Paeonia lactiflora* Pall. (*bái sháo*) 15g, the dried root of *Scutellaria baicalensis* Georgi (*huáng qín*) 15g, the dried root of *Platycodon grandiflorum* （Jacq）ADC（*jié gěng*） 12g, the dried root and rhizome of *Glycyrrhiza glabra* L. (*gān căo*) 10g, the dried rhizome of *Atractylodes macrocephala* Koidz. (*bái zhú*)15g, *Orthosiphon aristatus var. aristatus（shí gāo）* 20g, the dried root of *Stellaria dichotoma* L.var. lanceolata Bge（*yín chái hú*）12g, the dried ripe fruit of *Schisandra chinensis* (Turcz.) Baill. (*wŭ wèi zĭ*) 9g, the dried fruit of *Prunus mume* (Siebold) Siebold & Zucc. (*wū méi*) 9g, the dried velamen of *Dictamnus dasycarpus* Turcz (*bái xiān pí*) 15g, the dried ripe fruit of *Tribulus terrestris* L （jí lí） 15g. | Prepared by Quan et al |
| Bin Zhao (2021) | Xiaoxun decoction | The dry aboveground portion of *Schizonepeta tenuifolia* Briq （*jīng jiè*）15g, the dried root of *Saposhnikovia divaricata* （Turcz.）Schischk（*fáng fēng*）15g, the shell of *Cryptotympana pustulata* Fabricius (*chán tuì*)10g, the dried ripe fruit of *Tribulus terrestris* L （*jí lí*）15g, the dried root of *Angelica sinensis* (Oliv.) Diels (*dāng guī*) 6g, the dried rhizome of *Conioselinum anthriscoides 'Chuanxiong'* (*chuān xiōng*) 10g, the dried ripe peel of *Citrus × aurantium* L. (*chén pí*)10g, the dried sclerotium of *Poria cocos* (Schw.) Wolf (*fú líng*)10g, the dried rhizome of *Atractylodes macrocephala* Koidz. (*bái zhú*)10g, the dried root and rhizome of *Glycyrrhiza glabra* L. (*gān căo*)10g. | Prepared by Zhao et al |
| Shengbin Yu (2020) | Traditional Chinese Medicine fumigation | The dried root of*Arnebia euchroma*（Royle） Johnst or *Arnebia guttata* Bunge (*zǐ cǎo*) 30g, the dried root of *Saposhnikovia divaricata* （Turcz）Schischk（*fáng fēng*） 15g, the dried ripe fruit of *Kochia scoparia* (L.) Schrad (dì fū zǐ)30g, the ried rhizome of *Rehmannia glutinosa Libosch （dì huáng*） 30g, the dried ripe fruit of *Zanthoxylum schinifolium* Sieb. et Zucc（*huā jiāo*）10g, the dried velamen of *Dictamnus dasycarpus* Turcz (*bái xiān pí*)30g, the dried ripe fruit of *Cnidium monnieri*（L.）Cuss.（shé chuáng zǐ） 20g, the dried ripe fruit of *Prunus persica*（L.）Batsch（*táo rén*）30g, the dried root and rhizome of *Glycyrrhiza glabra* L. (*gān căo*) 15g, the dried root of *Paeonia lactiflora* Pall（*chì sháo*） 30g, *Cinnamomum camphora* (L.) J.Presl （*bīng piàn*） 5g, the dried root and rhizome of *Rheum palmatum L.* (*dà huáng*)30g, the dried root of *Sophora flavescens* Aiton (*kŭ shēn*)30g. | Prepared by Yu et al |
| Hanhua Cao (2019) | Chinese herbal fumigation | The dried root of *Saposhnikovia divaricata* （Turcz.）Schischk（*fáng fēng*） 15g, the shell of *Cryptotympana pustulata* Fabricius (*chán tuì*)15g, the dried rhizome of *Atractylodes macrocephala* Koidz. (*bái zhú*)50g, the dried root and rhizome of *Rheum palmatum L.* (*dà huáng*)30g, the dried root of *Smilax glabra* Roxb. (*tŭ fú líng*)30g, the dried ripe fruit of *Kochia scoparia* (L.) Schrad (*dì fū zǐ*)30g, the dried ripe fruit of *Cnidium monnieri*（L.）Cuss（*shé chuáng zǐ*） 30g, the dried rhizome of *Polygonum multiflorum* Thunb（*bái shǒu wū*）30g, the dried rhizome of *Spatholobus suberectus* Dunn（*jī xuè téng*）30g, the dried root of *Salvia miltiorrhiza* Bge（*dān shēn*）30g, the dried rhizome of *Conioselinum anthriscoides 'Chuanxiong'* (*chuān xiōng*) 30g, the dried root of *Sophora flavescens* Aiton (*kŭ shēn*) 10g, the dry aboveground portion of *Mentha haplocalyx* Briq（*bò he*）10g. | Prepared by Cao et al |
| Pengying Li (2019) | Traditional Chinese Medicine No.2 Prescription + Jianpi Jiedu Decoction | The dried root of *Smilax glabra* Roxb. (*tŭ fú líng*) 30g, the dried rhizome of *Dioscorea collettii* var. hypoglauca (Palib.) S.J. Pei & C.T. Ting (*bì xiè*)10g, the dried sclerotium of *Poria cocos* (Schw.) Wolf (*fú líng*)12g, the dried bark of *Phellodendron chinense* C.K. Schneid. (*huáng băi*) 20g, the dried ripe fruit of *Forsythia suspensa* （Thunb） Vahl（*lián qiào*）15g, the dried rhizome of *Atractylodes macrocephala* Koidz. (*bái zhú*)10g, the dried herb of *Scleromitrion diffusum* (Willd.) R.J. Wang (*bái huā shé shé căo*)30g, the dried root of *Angelica sinensis* (Oliv.) Diels (*dāng guī*) 20g, the dried root of *Salvia miltiorrhiza* Bge（*dān shēn*）10g, the dried root of *Sophora flavescens* Aiton (*kŭ shēn*)10g, the dried ripe kernel of *Coix lacryma-jobi* L. (*yì yĭ rén*) 20g, the dried root and rhizome of *Glycyrrhiza glabra* L. (*gān căo*) 10g + the dried root of *Smilax glabra* Roxb. (*tŭ fú líng*) 30g, the dried bark of *Phellodendron chinense* C.K. Schneid. (*huáng băi*)30g, the dried aboveground part of *Portulaca oleracea* L. (*mă chĭ xiàn*)30g, the dried ripe fruit of *Forsythia suspensa* （Thunb） Vahl（*lián qiào*） 30g, the dried rhizome of *Atractylodes macrocephala* Koidz. (*bái zhú*)30g, the dried sclerotium of *Poria cocos* (Schw.) Wolf (*fú líng*) 30g. | Prepared by Li et al |
| Shaoqun Qi (2019) | Jianpi Huashi decoction | The dried ripe peel of *Citrus × aurantium* L. (*chén pí*) 10g, the dried rhizome of *Atractylodes macrocephala* Koidz. (*bái zhú*)10g, the dried sclerotium of *Poria cocos* (Schw.) Wolf (*fú líng*) 10g, the dried stems and leaves of Lophatherum gracile Brongn (*dàn zhú yè*)10g, the dried root of *Saposhnikovia divaricata* （Turcz.）Schischk（*fáng fēng*） 10g, the dried velamen of *Dictamnus dasycarpus* Turcz(*bái xiān pí*) 10g, the dried rhizome of *Atractylodes lancea*（Thunb.）DC（*cāng zhú*） 10g, the dried ripe fruit of *Tribulus terrestris* L （*jí lí*） 10g, the dried ripe kernel of *Coix lacryma-jobi* L. (*yì yĭ rén*) 15g, the shells of *Ostrea gigas Thunberg(mǔ lì*)20g, the dried tuberous root of *Pseudostellaria heterophylla* (Miq.) Pax (*tài zĭ shēn*)20g, the dried root and rhizome of *Glycyrrhiza glabra* L. (*gān căo*) 6g. | Prepared by Qi et al |
| Yu Zhou (2017) | Liangxue Zhiyang decoction | The dried root of *Salvia miltiorrhiza* Bge（*dān shēn*）20g, the dried root of *Sophora flavescens* Aiton (*kŭ shēn*)12g, the dry aboveground portion of *Schizonepeta tenuifolia* Briq （*jīng jiè*）12g, the dried root of *Arnebia euchroma* (Royle ex Benth.) I.M. Johnst. (*zĭ căo*) 10g, the dried root of *Saposhnikovia divaricata* （Turcz）Schischk（*fáng fēng*）12g, the shell of *Cryptotympana pustulata* Fabricius (*chán tuì*) 8g, the dry flower heads of *Chrysanthemum indicum* L (*yě jú huā*) 10g, the dried ripe fruit of *Cnidium monnieri*（L.）Cuss（*shé chuáng zǐ*） 10g, the dried root of *Smilax glabra* Roxb. (*tŭ fú líng*)10g, the dry twigs of *Cinnamomum cassia* Presl (*guì zhī*)10g, the dried ripe fruit of *Ziziphus jujuba* Mill. (*dà zăo*) 12g, the dried root and rhizome of *Glycyrrhiza glabra* L. (*gān căo*) 6g. | Prepared by Zhou et al |
| Xiaojing Yang (2018) | White Tiger Decoction | The dried root of *Paeonia lactiflora* Pall*. (bái sháo)* 20g, *Orthosiphon aristatus var.* aristatus*（shí gāo）* 20g, the dry buds or with incipient flowers of *Lonicera japonica* Thunb（*jīn yín huā*） 20g, the dried root of *Scrophularia ningpoensis* Hemsl（*xuán shēn*） 20g, the dried whole grass *Taraxacum mongolicum* Hand. -Mazz *(pú gōng yīng)* 20g, the dried velamen of *Dictamnus dasycarpus* Turcz (*bái xiān pí*) 20g, the ried rhizome of *Rehmannia glutinosa Libosch （dì huáng*） 25g, the dried root of *Anemarrhena asphodeloides* Bge （zhī mǔ）15g, the died root bark of *Paeonia suffruticosa* Andr （*dān pí*） 15g, the died body of *Zaocys dhumnades* (Cantor) (*wū shāo shé*) 15g, the dried root of *Saposhnikovia divaricata* （Turcz）Schischk（*fáng fēng*）12g, the dried root of *Angelica sinensis* (Oliv.) Diels (*dāng guī*) 12g, the dry body of *Buthus martensii* Karsch (*quán xiē*)9g, the dried root of *Bupleurum chinense* DC（*chái hú*） 9g, the dried root and rhizome of *Glycyrrhiza glabra* L. (*gān căo*), 9g. | Prepared by Yang et al |
| Xiaohui Liu (2021) | Danggui Sini decoction | The dried root of *Angelica sinensis* (Oliv.) Diels (*dāng guī)* 30g, the dried rhizome of *Dioscorea oppositifolia* L. (*shān yào*)30g, the dried rhizome of *Conioselinum anthriscoides 'Chuanxiong'* (*chuān xiōng*) 15g, the dried rhizome of *Spatholobus suberectus* Dunn（*jī xuè téng*） 15g, the dried root and rhizome of *Glycyrrhiza glabra* L. (*gān căo*) 15g, the dried root of *Paeonia lactiflora* Pall （*chì sháo*）10g, the dried root of *Paeonia lactiflora* Pall. (*bái sháo*)10g, the dry aboveground portion of *Schizonepeta tenuifolia* Briq （*jīng jiè*）10g, the dried root of *Saposhnikovia divaricata* （Turcz）Schischk（*fáng fēng*） 6g, the dried ripe fruit of *Xanthium sibiricum* Patr（*cāng ěr zǐ*） 6g, the dried root of *Bupleurum chinense* DC（*chái hú*） 6g, the dry twigs of *Cinnamomum cassia* Presl (*guì zhī*)6g, the dried rhizome of *Asarum heterotropoides* Fr. Schmidt var. mandshuricum (Maxim.) Kitag（*xì xīn）* 3g. | Prepared by Liu et al |
| Tianming Ma（2）(2018) | Kushen Qufeng pills | - | Hospital system of the Second Affiliated Hospital of Heilongjiang University of Chinese Medicine agent |
| Yine Song (2017) | Yangxue Tongluo decoction | The dried root of *Smilax glabra* Roxb. (*tŭ fú líng*) 30g, the dried flower and bud of *Sophora japonica* L (*shēng huái huā*)20g, the dried root of *Angelica sinensis* (Oliv.) Diels (*dāng guī*)15g, the dried rhizome of *Spatholobus suberectus* Dunn（*jī xuè téng*）15g, the dried rhizome of *Clematis chinensis* Osbeck（*wēi líng xiān*） 15g, the dried root of *Saposhnikovia divaricata* （Turcz）Schischk（*fáng fēng*）15g, the ried rhizome of *Rehmannia glutinosa Libosch （dì huáng*）15g, the dried rhizome of *Ophiopogon japonicus* （L.f）Ker-Gawl （*mài dōng*） 10g. | Prepared by Song et al |
| Ying Yang (2006) | Jianpi Zhiyang Granules | The dried root of *Astragalus mongholicus* Bunge (*huáng qí*)*,* the dried rhizome of *Atractylodes macrocephala* Koidz. (*bái zhú*)*,* the dried root of *Angelica sinensis* (Oliv.) Diels (*dāng guī*)*,* the dried rhizome of *Polygonum multiflorum* Thunb (*shǒu wū*）*,*the ried rhizome of *Rehmannia glutinosa Libosch （dì huáng*）*,* the dried root of *Paeonia lactiflora* Pall. (*bái sháo*)*, t*he dried rhizome of *Conioselinum anthriscoides 'Chuanxiong'* (*chuān xiōng*)*,* the dry aboveground portion of *Schizonepeta tenuifolia* Briq （*jīng jiè*）*,* the dried root of *Saposhnikovia divaricata* （Turcz）Schischk（*fáng fēng*）. | Hospital preparation of Shenzhen Hospital of Traditional Chinese Medicine, Affiliated Hospital of Guangzhou University of Chinese Medicine |
| Saiqian Hu (2018) | Modified Siwu Decoction Combined with Yangxue Runzao Zhiyang Prescription | The dried root of *Angelica sinensis* (Oliv.) Diels (*dāng guī*)10g, the dried rhizome of *Conioselinum anthriscoides 'Chuanxiong'* (*chuān xiōng*) 10g, the dried root of *Paeonia lactiflora* Pall. (*bái sháo*) 10g, the ried rhizome of *Rehmannia glutinosa Libosch （dì huáng*） 15g, the dried velamen of *Dictamnus dasycarpus* Turcz (*bái xiān pí*) 10g, the dried root of *Saposhnikovia divaricata* （Turcz）Schischk（*fáng fēng*） 10g, the dry aboveground portion of *Schizonepeta tenuifolia* Briq （*jīng jiè*）10g, the dried root of *Smilax glabra* Roxb. (*tŭ fú líng*)20g, the dried ripe fruit of *Kochia scoparia* (L.) Schrad (*dì fū zǐ*)*2*0g, the dried ripe fruit of *Ligustrum lucidum* Ait（*nǚ zhēn zǐ*）15g, the dried root of *Sophora flavescens* Aiton (*kŭ shēn*) 15g, the dried root of *Anemarrhena asphodeloides* Bge （*zhī mǔ*）15g, the dry aboveground portion of *Eclipta prostrata* L（*mò hàn lián*）20g, the dried bark of *Phellodendron chinense* C.K. Schneid. (*huáng băi*)12g, the died root bark of *Paeonia suffruticosa* Andr （mǔ dān pí）12g, the horn of *Bubalus bubalis* Linnaeus (*shuǐ niú jiǎo*)10g. | Prepared by Hu et al |
| Lan Ding (2021) | Yangxue Qufeng External Washing Prescription | The dried root of *Angelica sinensis* (Oliv.) Diels (*dāng guī)* 15g, the dried root of *Paeonia lactiflora* Pall （*chì sháo*）10g, the dried rhizome of *Conioselinum anthriscoides 'Chuanxiong'* (*chuān xiōng*) 9g, the died root bark of *Paeonia suffruticosa* Andr （*mǔ dān pí*） 15g, the dried root of *Sophora flavescens* Aiton (*kŭ shēn*)15g, the dried root of *Smilax glabra* Roxb. (*tŭ fú líng*) 15g, the dried ripe fruit of *Tribulus terrestris* L （*jí lí*）15g, the dried stems of *Polygonum multiflorum* Thunb (*yè jiāo téng*) 30g, the dried ripe fruit of *Kochia scoparia* (L.) Schrad (*dì fū zǐ*)15g, the dried ripe fruit of *Cnidium monnieri*（L.）Cuss（*shé chuáng zǐ*）15g, the dry aboveground portion of *Schizonepeta tenuifolia* Briq （*jīng jiè*） 10g, the dried root of *Saposhnikovia divaricata* （Turcz）Schischk（*fáng fēng*） 10g, the dried root and rhizome of *Glycyrrhiza glabra* L. (*gān căo*) 30g. | Prepared by Ding et al |
| Yinping Ma (2018) | Yangxue Zhiyang Decoction | The ried rhizome of *Rehmannia glutinosa* Libosch *（dì huáng*）25g, the dried rhizome of *Asparagus cochinchinensis*（Lour.）Merr（*tiān dōng*）25g, the dried rhizome of *Ophiopogon japonicus* （L.f）Ker-Gawl （*mài dōng*） 25g, the died root bark of *Paeonia suffruticosa* Andr （*mǔ dān pí*） 20g, the dry aboveground portion of *Schizonepeta tenuifolia* Briq （*jīng jiè*） 20g, the dried root of *Angelica sinensis* (Oliv.) Diels(*dāng guī*)10g, the dried ripe fruit of *Tribulus terrestris* L （*jí lí*）10g, the shell of *Cryptotympana pustulata* Fabricius (*chán tuì*)10g, the dried root and rhizome of *Glycyrrhiza glabra* L. (*gān căo*) 10g. | Prepared by Ma et al |
| Mehrzad Mehrbani | extract of field dodder | dodder seed | Prepared by Mitra Mehrabani et al |
| Qing Wu (2020) | Qingre Liangxue decoction | The dried ripe fruit of *Forsythia suspensa* （Thunb.） Vahl（*lián qiào*） 30g, the dried root of *Arnebia euchroma* (Royle ex Benth.) I.M. Johnst. (*zĭ căo*)25g, the horn of *Bubalus bubalis* Linnaeus (*shuǐ niú jiǎo*)15g, the dry buds or with incipient flowers of *Lonicera japonica* Thunb（*jīn yín huā*） 15g, the died root bark of *Paeonia suffruticosa* Andr （*mǔ dān pí*）12g, the ried rhizome of *Rehmannia glutinosa Libosch （dì huáng*） 12g, the dried root of *Scutellaria baicalensis* Georgi (*huáng qín*) 10g, the dried root of *Paeonia lactiflora* Pall （*chì sháo*）10g, the dried flower of *Campsis grandiflora*（Thunb.）K.Schum（*líng xiāo huā*）10g, the dried root and rhizome of *Glycyrrhiza glabra* L. (*gān căo*) 9g. | Prepared by Wu et al |
| Rui Tao (2020) | Jiangtang Huoxue Prescription combined with Xiaofeng Powder | The dried rhizome of *Atractylodes lancea*（Thunb）DC（*cāng zhú*） 15g, the dried root of *Scrophularia ningpoensis* Hemsl（*xuán shēn*） 20g, the dried root of *Pueraria lobata* (Willd.) Ohwi (*gé gēn*)15g, the dried root and rhizome of *Salvia miltiorrhiza* Bunge (*dān shēn*) 20g, the dried root of *Astragalus mongholicus* Bunge (*huáng qí*) 20g, the ried rhizome of *Rehmannia glutinosa Libosch （dì huáng*） 20g, the dried root of *Dolomiaea costus* (Falc.) Kasana & A.K. Pandey (*mù xiāng*)10g, the dried root of *Angelica sinensis* (Oliv.) Diels (*dāng guī*) 10g, the dry aboveground portion of *Leonurus japonicus* Houtt（*yì mǔ cǎo*）15g, the dried root of *Paeonia lactiflora* Pall. (*bái sháo*) 15g, the dried rhizome of *Conioselinum anthriscoides 'Chuanxiong'* (*chuān xiōng*) 10g, the dried root of *Saposhnikovia divaricata* （Turcz.）Schischk（*fáng fēng*）10g, the shell of *Cryptotympana pustulata* Fabricius (*chán tuì*)10g, the dried root of *Anemarrhena asphodeloides* Bge （*zhī mǔ*）10g, the dried root of *Sophora flavescens* Aiton (*kŭ shēn*)6g, the dried rhizome of *Akebia quinata* （Thunb.）Decne（*mù tōng*） 6g, the dry aboveground portion of *Schizonepeta tenuifolia* Briq （*jīng jiè*） 10g, the dried ripe fruit of *Arctium lappa* L（*niú bàng zǐ*）10g, *Orthosiphon aristatus var.* aristatus（*shí gāo*） 15g, the dried root and rhizome of *Glycyrrhiza glabra* L. (*gān căo*) 6g. | Prepared by Tao et al |
| Xinwei Guo (2020) | Modified Chushi Weiling Decoction | The dried rhizome of *Atractylodes lancea*（Thunb.）DC（*cāng zhú*）6g, the died root bark of *Magnolia officinalis* Rehd.et Wils（*hòu pǔ*） 6g, the dried ripe peel of *Citrus × aurantium* L. (*chén pí*)9g, *Orthosiphon aristatus var*. aristatus （*huá shí*）12g, the dried rhizome of *Atractylodes macrocephala* Koidz. (*bái zhú*) 12g, the dried sclerotium of *Polyporus umbellatus*（Pers.） Fries （*zhū líng*） 12g, the dried bark of *Phellodendron chinense* C.K. Schneid. (*huáng băi*) 12g, the dried unripe fruit of *Citrus × aurantium* L. (*zhĭ qiào*) 9g, the dried rhizome of *Alisma orientale*（Sam.）Juzep.or*Alisma plantago*-aquatica Linn. ( *zé xiè*)9g, the dried sclerotium of *Poria cocos* (Schw.) Wolf (*fú líng*) 12g, the dried root and rhizome of *Glycyrrhiza glabra* L. (*gān căo*) 9g. | Prepared byGuo et al |
