# Supplementary figures and images for: Efficacy and safety of Chinese herbal medicine in the treatment of chronic pruritus: A systematic review and meta-analysis of randomized controlled trials
Source: Front Pharmacol. 2023 Jan 12;13:1029949. doi: 10.3389/fphar.2022.1029949 (PMC9877228; doi:10.3389/fphar.2022.1029949)

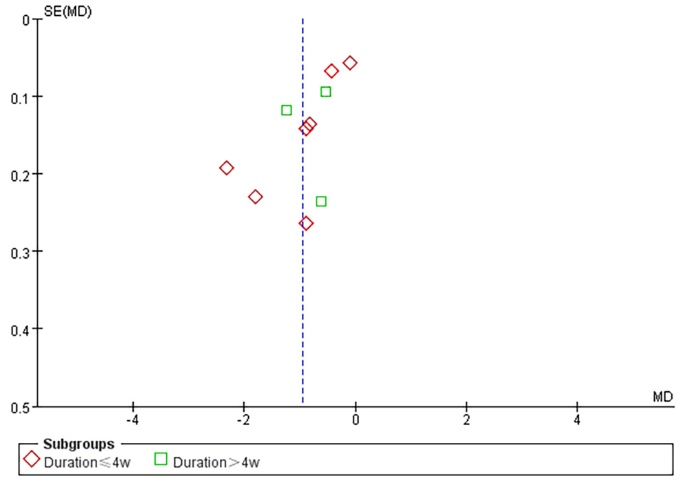


Figure S1: The funnel plot of pruritus.

Supplement: Supplementary file 2 [file DataSheet1.docx]
